# Predicting breast cancer 5-year survival using machine learning: A systematic review

**DOI:** 10.1371/journal.pone.0250370

**Published:** 2021-04-16

**Authors:** Jiaxin Li, Zijun Zhou, Jianyu Dong, Ying Fu, Yuan Li, Ze Luan, Xin Peng

**Affiliations:** 1 School of Nursing, Jilin University, Jilin, China; 2 Breast Surgery, Jilin Province Tumor Hospital, Jilin, China; Medical University of Vienna, AUSTRIA

## Abstract

**Background:**

Accurately predicting the survival rate of breast cancer patients is a major issue for cancer researchers. Machine learning (ML) has attracted much attention with the hope that it could provide accurate results, but its modeling methods and prediction performance remain controversial. The aim of this systematic review is to identify and critically appraise current studies regarding the application of ML in predicting the 5-year survival rate of breast cancer.

**Methods:**

In accordance with the PRISMA guidelines, two researchers independently searched the PubMed (including MEDLINE), Embase, and Web of Science Core databases from inception to November 30, 2020. The search terms included breast neoplasms, survival, machine learning, and specific algorithm names. The included studies related to the use of ML to build a breast cancer survival prediction model and model performance that can be measured with the value of said verification results. The excluded studies in which the modeling process were not explained clearly and had incomplete information. The extracted information included literature information, database information, data preparation and modeling process information, model construction and performance evaluation information, and candidate predictor information.

**Results:**

Thirty-one studies that met the inclusion criteria were included, most of which were published after 2013. The most frequently used ML methods were decision trees (19 studies, 61.3%), artificial neural networks (18 studies, 58.1%), support vector machines (16 studies, 51.6%), and ensemble learning (10 studies, 32.3%). The median sample size was 37256 (range 200 to 659820) patients, and the median predictor was 16 (range 3 to 625). The accuracy of 29 studies ranged from 0.510 to 0.971. The sensitivity of 25 studies ranged from 0.037 to 1. The specificity of 24 studies ranged from 0.008 to 0.993. The AUC of 20 studies ranged from 0.500 to 0.972. The precision of 6 studies ranged from 0.549 to 1. All of the models were internally validated, and only one was externally validated.

**Conclusions:**

Overall, compared with traditional statistical methods, the performance of ML models does not necessarily show any improvement, and this area of research still faces limitations related to a lack of data preprocessing steps, the excessive differences of sample feature selection, and issues related to validation. Further optimization of the performance of the proposed model is also needed in the future, which requires more standardization and subsequent validation.

## Introduction

Breast cancer is the most common cancer among women in 154 countries and the main cause of cancer-related death in 103 countries. In 2018, there were approximately 2.1 million new cases of breast cancer in women, accounting for 24.2% of the total cases, and the mortality rate was approximately 15.0% [1].

Survival is defined as the period of time a patient survives after disease diagnosis.The 5-year threshold is important to standardize reporting and to identify survivability. Labelling a patient record as survived or not survived takes at least 5 years, therefore, some previous studies used a 5-year threshold to identify the cohort’s survivability [2]. Breast cancer is a complex disease, and although its survival rates in recent years have increased gradually, its 5-year survival rate is considerably different between individuals [3]. Predicting breast cancer survival accurately could help doctors make better decisions regarding medical treatment intervention planning, prevent excessive treatment, thereby reducing economic costs [4, 5], more effectively include and exclude patients in a randomized trial [6], and develop palliative care and hospice care systems [7, 8]. Therefore, predicting survival has become a major issue in current research on breast cancer.

With the surge of medical data as well as the rapid development of information technology and artificial intelligence, the application of big data analysis technology in the construction of survival prediction model has become a current research hotspot. Traditional prediction models based on prior hypothesized knowledge often consider the relationship between dependent variables; in contrast, ML has the potential of learning data models automatically, does not require any implicit assumptions and is able to handle interdependence and nonlinear relationships between variables [9]. It has natural strengths in dealing with the very large number of complex higher-order interactions of medical data. Therefore ML tools have a high potential for application in routine medical practice as leading tools in health informatics.

A growing number of ML studies have been applied to diagnosis [10–13], disease risk prediction [14], recurrence prediction [15], and symptom prediction [16–19]. Furthermore, although the number of survival predictions increases gradually, the database set, modeling process, methodological quality, performance metrics, and modeling of related candidate predictors exhibit large differences [20].

This article aims to systematically and comprehensively review the published literature regarding the use of ML algorithms for model development and validation of breast cancer survival prediction. The primary outcome indicator is the accuracy of the different models in predicting 5-year (60 months or 1825 days) survival rate for breast cancer with the goal of providing a better theoretical basis for the application of ML in survival prediction.

## Methods

### Trial registration

This research was registered in the International Prospective Register of Systematic Reviews (PROSPERO) in November 2020 (CRD42020219154). https://www.crd.york.ac.uk/PROSPERO/#recordDetails.

### Search strategy

This research was conducted in accordance with the Preferred Reporting Items for Systematic Reviews and Meta-Analysis (PRISMA) guidelines [20] (see S1 Table). Two researchers (Jiaxin Li and Jianyu Dong) independently searched PubMed (including MEDLINE) (1966~present), Embase (1980~present), and Web of Science Core Collection (1900~present) databases from inception to November 30, 2020. EndNote X9 software was used to remove duplicate literature. Detailed search strategies are listed in the (see S2 Table).

### Inclusion and exclusion criteria

The inclusion criteria were as follows: (1) published peer-reviewed literature; (2) research on the clinical diagnosis of breast cancer patients; (3) research related to the use of ML algorithms to build a survival prediction model; (4) prediction models established through the internal or external validation; (5) model performance that can be measured with the value of said verification results; and (6) studies published in English.

The exclusion criteria were as follows: (1) studies in which the training, learning, and/or validation process were not explained clearly or distinguished from each other; (2) duplicate studies; (3) literature reviews; (4) non-human (e.g., animals) studies; (5) case reports; (6) expert experience reports; and (7) unavailable full text or incomplete abstract information such that effective information cannot be extracted.

### Data extraction

Two researchers (Jiaxin Li and Jianyu Dong) independently screened and cross-checked the documents to extract information. If there were differences in the process, then a third party was consulted (Ying Fu). MS office Excel 2019 software was used for basic information literature screening. First, the titles and abstracts were screened to exclude unrelated literature; then, the full texts of articles were read to determine their eligibility for inclusion. The Checklist for critical Appraisal and data extraction for systematic Reviews of prediction Modelling Studies (CHARMS) was also used for data extraction [21], the extracted data include the following:

- Basic literature information: first author, year, country of research, published type, disease characters, and predicted outcome;
- Basic data information: data source, data type, number of centers, and number of samples;
- Data preparation and modeling process information: missing data described, missing data processing described, preprocessing algorithms and preprocessing described, feature selection algorithms and feature selection described, class imbalance (Alive + Dead), number of candidate predictors used, ML algorithms, model presentation, and software or environment used;
- Model construction and performance evaluation information: internal validation, external validation, model evaluation metrics, calibration metrics, hyperparameter tuning, and discrimination and classification metrics; and
- Candidate predictor information: number of candidate predictors, candidate predictors, process for ranking of candidate predictors, and rank of candidate predictors.

### Assessment of the risk of bias

Two researchers used the prediction model risk of bias assessment tool (PROBAST) [22]. PROBAST is mainly used in research and development validation or to update multivariate predictor diagnosis or prognosis prediction models. The tool includes 20 signaling questions across 4 domains (participants, predictors, outcome, and analysis), and each question is answered as low risk of bias assessment, high risk of bias assessment, or unclear.

## Results

### Search results

**Fig 1:**
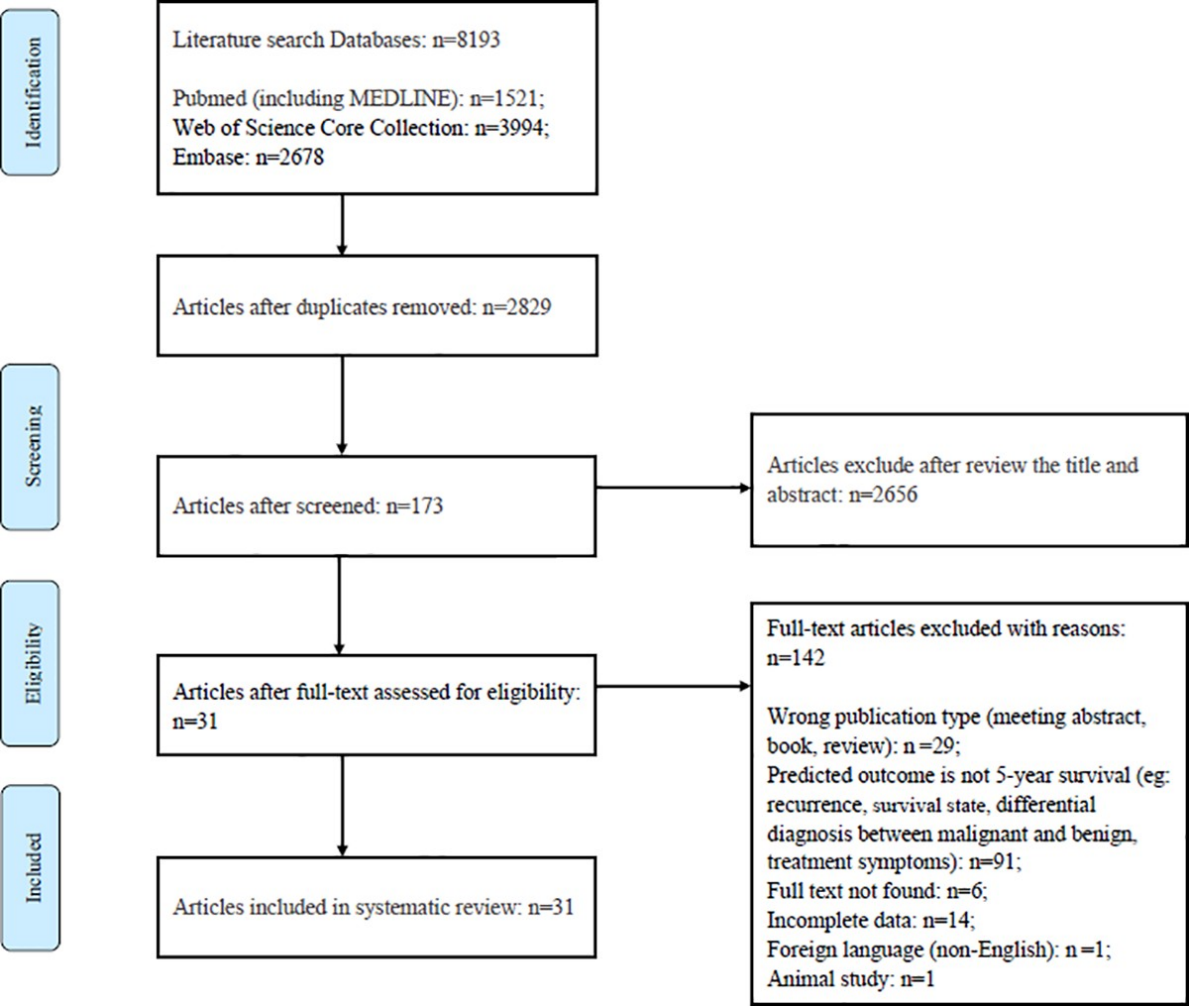
PRISMA flowchart.

By searching three medical databases, a total of 8193 studies were identified. After removing duplicates studies. there were leaving 2829 studies and 2656 studies were eliminated based on the screening of titles and abstracts. A comprehensive review of the full text of the remaining 173 studies was conducted, and 142 were excluded for the following reasons: the type of literature did not meet the criteria, i.e., conference abstracts, books, and review literature (n = 9); the predictive outcome was not 5-year survival but recurrence, survival status, benign and malignant tumor diagnosis, or treatment symptoms (n = 91); the full text was unavailable (n = 6); the data were incomplete (n = 14); the study was not published in English (n = 1); or the study included animal research (n = 1). A total of 31 studies met the inclusion criteria [2, 23–52]. The literature screening process is shown in Fig 1.

### Assessment of the risk of bias

Among the 31 studies, 9 had a high risk of bias [2, 25, 27, 28, 43, 44, 46, 48, 50], 17 had a moderate risk of bias [24, 26, 29–35, 39, 41, 42, 45, 47, 49, 51, 52], and 5 ad a low risk of bias [23, 36–38, 40], as shown in Table 1.

**Table 1:** Risk of bias and applicability assessment grading of 31 studies as per the PROBAST criteria.

Research	Participant bias	Predictor bias	Outcome bias	Analysis bias	Overall bias rating	Overall applicability rating
**Delen, 2005 [2]**	Low	Low	Low	High	High	Low
**Bellaachia, 2006 [23]**	Low	Low	Low	Low	Low	Low
**Endo, 2007 [24]**	Low	Low	Low	Moderate	Moderate	Low
**Khan, 2008 [25]**	Low	Low	Low	High	High	Low
**Thongkam, 2008** [26]	Low	Low	Low	Moderate	Moderate	Low
**Choi, 2009 [27]**	Low	Low	Low	High	High	Low
**Liu, 2009 [28]**	Low	Low	Low	High	High	Low
**Wang, 2013 [29]**	Low	Low	Low	Moderate	Moderate	Low
**Kim, 2013 [30]**	Low	Low	Low	Moderate	Moderate	Low
**Park, 2013 [31]**	Low	Low	Low	Moderate	Moderate	Low
**Shin, 2014 [32]**	Low	Low	Low	Moderate	Moderate	Low
**Wang, 2015 [33]**	Low	Low	Low	Moderate	Moderate	Low
**Wang, 2014 [34]**	Low	Low	Low	Moderate	Moderate	Low
**Chao, 2014 [35]**	Low	Low	Low	Moderate	Moderate	Low
**García-Laencina, 2015 [36]**	Low	Low	Low	Low	Low	Low
**Lotfnezhad Afshar, 2015 [37]**	Low	Low	Low	Low	Low	Low
**Khalkhali, 2016 [38]**	Low	Low	Low	Low	Low	Low
**Shawky, 2016 [39]**	Low	Low	Low	Moderate	Moderate	Low
**Sun, 2018 [40]**	Low	Low	Low	Low	Low	Low
**Sun, 2018 [41]**	Low	Low	Low	Moderate	Moderate	Low
**Zhao, 2018 [42]**	Low	Low	Low	Moderate	Moderate	Low
**Fu, 2018 [43]**	Low	Low	High	High	High	High
**Lu, 2019 [44]**	Low	Low	Low	High	High	Low
**Abdikenov, 2019 [45]**	Low	Low	Low	Moderate	Moderate	Low
**Kalafi, 2019 [46]**	Low	Low	Low	High	High	Low
**Shouket, 2019 [47]**	Low	Low	Low	Moderate	Moderate	Low
**Ganggayah, 2019 [48]**	Low	Low	Low	High	High	Low
**Simsek, 2020 [49]**	Low	Low	Low	Moderate	Moderate	Low
**Salehi, 2020 [50]**	Low	Low	Low	High	High	Low
**Tang, 2020 [51]**	Low	Moderate	Low	Moderate	Moderate	Moderate
**Hussain, 2020 [52]**	Low	Low	Low	Moderate	Moderate	Low

### Primary characteristics of the literature

**Fig 2:**
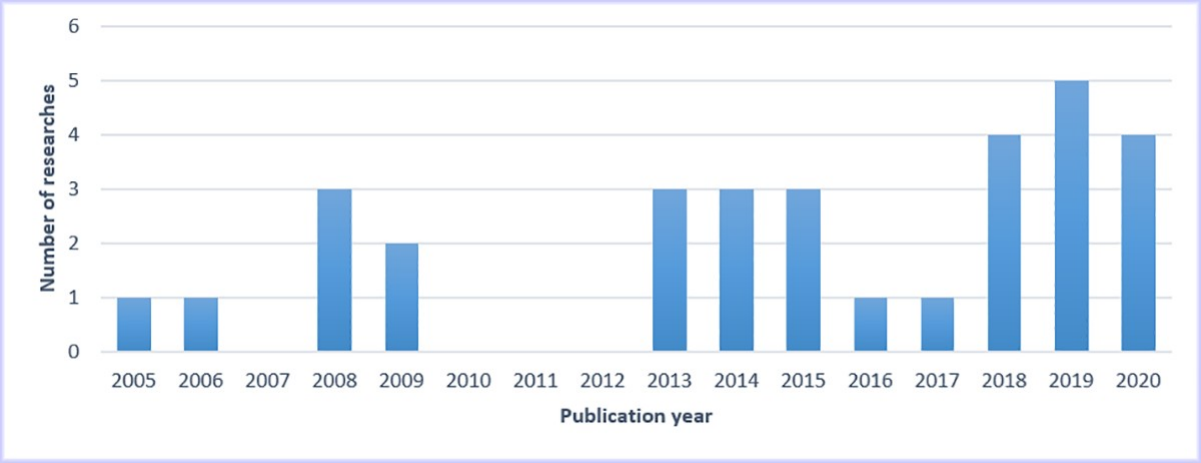
Number of studies published each year.

The primary characteristics of the 31 studies are shown in Table 2. Most of the 31 studies were published from 2013 to 2020, and the statistics regarding the publication year and number of studies are shown in Fig 2. Among them, 22 studies were located in Asia [24, 25, 27–35, 37, 38, 40, 41, 43, 46–48, 50–52], 5 in North America [2, 23, 42, 44, 49], 2 in Oceania [26, 45], 1 in Europe [36], and 1 in Africa [39]. The primary prediction outcome was the 5-year survival of breast cancer patients. The predicted disease types were all breast cancer rather than one particular subtype (e.g., triple-negative breast cancer). All included studies focused on the development of survival prediction models using ML algorithms rather than validating the existing models on independent data. Primary characteristics are shown in Tables 2 and 3.

**Table 2:** Primary characteristics and details of the 31 studies (Table 2 was uploaded as an additional file).

First author, year	Country of research	Source of data	Number of samples (Alive+ Dead)	Missingness processing described	Preprocessing described	Feature selection described	Number of candidate predictors used	ML algorithms	Validation method	Model evaluation metrics	Hyperparameter tuning
**Delen, 2005 [2]**	USA	SEER (1973–2000)	202932 (109659+93273)	Yes	Yes	No	16	ANN; C5 DT; LR	Internal validation	Accuracy; Sensitivity; Specificity	No
**Bellaachia, 2006 [23]**	USA	SEER (1973–2002)	151886 (116738+35148)	No	Yes	No	16	Naive Bayes; MLP; C4.5 DT	Internal validation	Accuracy; Sensitivity (Recall); Precision	No
**Endo, 2008 [24]**	Japan	SEER (1972–1997)	37256 (30734+6882)	No	Yes	No	10	LR; ANN; Naive Bayes; Bayesian network; DT + Naive Bayes; DT ID3; DT J48	Internal validation	Accuracy; Sensitivity; Specificity	No
**Khan, 2008 [25]**	Korea	SEER (1973–2003)	162500 (unknown)	Yes	Yes	No	16	Fuzzy decision trees	Internal validation	Accuracy; Sensitivity; Specificity	No
**Thongkam, 2008 [26]**	Australia	Srinagarind Hospital records in Thailand (1990–2001)	732 (342+394)	No	Yes	No	11	C4.5 DTs; Conjunctive rule; Naive Bayes; NN-classifier; random committee; RF; Radial basis function network	Internal validation	Accuracy; AUC	No
**Choi, 2009 [27]**	Korea	SEER (1973–2003)	294275 (unknown)	Yes	Yes	No	14	ANN; Bayesian network; Hybrid Bayesian network	Internal validation	Accuracy; Sensitivity; Specificity; AUC	No
**Liu, 2009 [28]**	China	SEER (1973–2004)	182517 (157916+24601)	Yes	Yes	Yes	16	C5 DT	Internal validation	Accuracy; Sensitivity; Specificity; AUC	No
**Wang, 2013 [29]**	China	SEER (1973–2007)	215221 (195172+20049)	Yes	Yes	Yes	9	DT; LR	Internal validation	Accuracy; Sensitivity; Specificity; AUC; G-mean	No
**Kim, 2013 [30]**	Korea	SEER (1973–2003)	162500 (128469+34031)	No	Yes	No	16	SVM; ANN; Semi-supervised learning; Semi-supervised learning-Co training	Internal validation	Accuracy; AUC	No
**Park, 2013 [31]**	Korea	SEER (1973–2003)	162500 (128469+34031)	No	Yes	No	16	SVM; ANN; Semi-supervised learning	Internal validation	Accuracy; Sensitivity; Specificity; AUC	Yes
**Shin, 2014 [32]**	Korea	SEER (1973–2003)	162500 (128469+34031)	No	Yes	No	16	DT; ANN; SVM; Semi-supervised learning; Semi-supervised learning-Co training	Internal validation	AUC	Yes
**Wang, 2015 [33]**	China	Northern Taiwan hospital database (2000–2003)	604 (464+140)	Yes	Yes	Yes	5	ANN	Internal validation	Accuracy; Sensitivity; Specificity; AUC	No
**Wang, 2014 [34]**	China	SEER (1973–2007)	215221 (195172+20049)	No	Yes	No	20	LR; C5 DT; 1-nearest neighbor	Internal validation	Accuracy; Sensitivity; Specificity; G-mean	Yes
**Chao, 2014 [35]**	China	Breast cancer incidence database in Taiwan hospital (2002–2010)	1340 (1272+68)	No	Yes	No	7	SVM; LR; C5 DT	Internal validation	Accuracy	Yes
**García-Laencina, 2015 [36]**	Spain	Institute Portuguese of Oncology of Porto	399 (282+117)	Yes	Yes	No	16	KNN; Classification Trees; LR; SVM	Internal validation	Accuracy; Sensitivity; Specificity; AUC	Yes 2-D grid search
**Lotfnezhad Afshar, 2015 [37]**	Iran	SEER (1999–2004)	22763 (Aliveness values were approximately nine times greater than death values)	Yes	Yes	No	18	SVM; Bayesian network; Chi-squared Automatic Interaction Detection	Internal validation	Accuracy; Sensitivity; Specificity; Adjusted propensity	No
**Khalkhali, 2016 [38]**	Iran	Omid Treatment and Research Center database (2007–2010)	569 (unknown)	Yes	Yes	No	15	Classification and regression tree	Internal validation	Accuracy; Sensitivity; Specificity	No
**Shawky, 2017 [39]**	Egypt	SEER (2010)	4490 (2245+2245)	Yes	Yes	No	14	ANN; KNN; SVM; LR	Internal validation	Accuracy; Sensitivity; Specificity; AUC	No
**Sun, 2018 [40]**	China	Molecular Taxonomy of Breast Cancer International Consortium	1980 (1489+491)	Yes	Yes	Yes	3 types	SVM; RF; LR; DNN	Internal and external validation	Accuracy; Sensitivity; Precision; Specificity; AUC; Mcc	Yes
**Sun, 2018 [41]**	China	The Cancer Genome Atlas	578 (133+445)	No	Yes	Yes	5 types	Multiple kernel learning; Regular Cox models; Parametric censored regression models; Random survival forests; Boosting concordance index; Supervised principal components regression	Internal validation	Accuracy; Sensitivity; Specificity; Precision; AUC; Mcc; C-index	No
**Zhao, 2018 [42]**	USA	Molecular Taxonomy of Breast Cancer International Consortium	1874 (1409+465)	Yes	Yes	No	27	Gradient Boosting; RF; SVM; ANN	Internal validation	Accuracy; AUC	No
**Fu, 2018 [43]**	China	The Clinical Research Center for Breast in West China Hospital of Sichuan University (1989–2007)	5246 (1181+4065)	Yes	Yes	Yes	23	Gradient Boosting; DT framework; SVM; RF; Adaboost; Cox Regression	Internal validation	Cutoff; Youden index; Sensitivity; F-score; Specificity; AUC	Yes TPE algorithm
**Lu, 2019 [44]**	USA	SEER (1973–2014)	82707 (76716+5991)	Yes	Yes	No	14	Genetic algorithm-based online gradient; Online Sequential Extreme Learning Machine; Online Adaptive Boosting with the Adaptive Linear Regressor; Online Gradient Boosting with the Adaptive Linear Regressor; Online linear regressor; AdaBoost; SVM; MLP	Internal validation	Accuracy; Sensitivity; Specificity; AUC; Retraining time	Yes
**Abdikenov, 2019 [45]**	Australia	SEER (2004–2014)	659802 (376087+283715)	No	Yes	No	19	DNN; LR; SVM; RF; Gradient Boosting	Internal validation	Accuracy; Sensitivity; Specificity; AUC; F1 score	Yes NSGAIII
**Kalafi, 2019 [46]**	Malaysia	The University Malaya Medical Centre Breast Cancer Registry database (1993–2017)	4902 (2451+2451)	Yes	Yes	No	23	SVM; RF; DT; MLP	Internal validation	Accuracy; Sensitivity; Precision; Specificity; F1 score; Mcc; NPV; FPR; FDR; FNR	No
**Shouket, 2019 [47]**	Pakistan	Institute of Nuclear Medicine & Oncology Lahore Hospital of Pakistan database (2013–2018)	200 (5-year survival: 190+10; 5-year disease free survival: 164+36)	No	Yes	No	10	Naive Bayes; DT J48; SVM; RF; AdaBoost; JRip	Internal validation	Accuracy; Precision; AUC; F-score; Mcc; NPV	No
**Ganggayah, 2019 [48]**	Malaysia	The University Malaya Medical Centre Breast Cancer Registry database (1993–2017)	8066 (5614+2452)	Yes	Yes	Yes	23	DT (rpart); RF; ANN; XGboost; LR; SVM	Internal validation	Accuracy; Sensitivity; Specificity; AUC; Precision; Mcc; Calibration curve	No
**Simsek, 2020 [49]**	USA	SEER (1973–2013)	53732 (1-year: 52886+886; 5-year: 46724+7028; 10-year: 42965+10787.)	Yes	Yes	Yes	17	ANN; LR	Internal validation	Accuracy; Sensitivity; Specificity; AUC	No
**Salehi, 2020 [50]**	Iran	SEER (2004–2013)	141254 (118324+22930)	Yes	Yes	No	35	MLP; MLP experts; MLP stacked generalization	Internal validation	Accuracy; Sensitivity; Specificity	No
**Tang, 2020 [51]**	China	Haberman’s Survival Data Set (1958–1970)	306 (unknown)	No	Yes	No	3	Evolutionary dendritic neuron model; MLP; MLP with adaptive learning rate and momentum coefficient; DT; SVM trained by the radial basis function kernel; SVM trained by the linear kernel; SVM trained by the polynomial kernel; Dendritic neuron model	Internal validation	Accuracy; Sensitivity; Specificity; AUC; F-score	Yes
**Hussain, 2020 [52]**	Iraq	SEER (1973–2001)	90308 (unknown)	Yes	Yes	No	17	ANN; DT; LR	Internal validation	Accuracy; Sensitivity; Specificity	No

**Table 3:** Primary characteristics and categories of the 31 studies.

Characteristics	Categories	Number (n)	Percentage (%)
**Place of research**	Asia	22	71.0
	North America	5	16.1
	Oceania	2	6.5
	Europe	1	3.2
	Africa	1	3.2
**Published type**	Journal article	27	87.1
	Conference paper	3	9.6
	Information paper	1	3.2
**Source of data**	SEER	18	58.1
	Molecular Taxonomy of Breast Cancer International Consortium	2	6.5
	The Cancer Genome Atlas	1	3.2
	Haberman’s Cancer Survival Dataset	1	3.2
	Hospital Registration Data	9	29.0
**Type of data**	Public	22	71.0
	Private	9	29.0
**Number of centers**	Single center	22	71.0
	Multiple centers	9	29.0
**Sample size**	<1000	7	22.6
	1000~10000	7	22.6
	>10000	17	54.8
**Missing data and processing described**	Yes	20	64.5
	No	11	35.3
**Preprocessing described**	Yes	31	100.0
	No	0	0.0
**Feature selection described**	Yes	8	25.8
	No	23	74.2
**Class imbalance processing**	Yes	24	77.4
	No	2	6.5
	Unknown	5	16.1
**Number of candidate predictors**	<10	4	12.9
	10~100	25	80.6
	>100	2	6.5
**Number of ML algorithms**	1	5	16.1
	>1	26	83.9
**Type of ML algorithms**	DT	19	61.3
	ANN	18	58.1
	SVM	16	51.6
	LR	12	38.7
	Bayesian classification algorithms	6	19.4
	KNN	3	9.7
	Semi-supervised learning	3	9.7
	Ensemble learning	10	32.3
	DNN	3	9.7
**Model presentation**	Formula	6	19.4
	Graph	5	16.1
	Formula and graph	16	51.6
	No presentation	4	12.9
**Calibration**	Yes	1	3.2
	No	30	96.8
**Internal validation**	Yes	31	100.0
	No	0	0.0
**External validation**	Yes	1	3.2
	No	30	96.8
**Hyperparameter selection**	Yes	9	29.0
	No	22	71.0
**Model evaluation metrics**	Accuracy	29	93.5
	Sensitivity/Recall	25	80.6
	Specificity	24	77.4
	AUC	20	64.5
	Precision/Positive predictive value	6	19.4
	F1 score	5	16.1
	Mcc	5	16.1
	NPV	2	6.5
	G-mean	2	6.5
	C-index	1	3.2
	Cutoff	1	3.2
	Youden index	1	3.2
	Retaining time	1	3.2
	FPR	1	3.2
	FDR	1	3.2
	FNR	1	3.2
**Type of candidate predictors**	Clinical data	29	93.5
	Clinical data + molecular data	1	3.2
	Clinical data + molecular data + pathological images	1	3.2
**Ranking of candidate predictors**	Yes	15	28.4
	No	16	51.6

### Primary database information

Eighteen studies used the SEER database [2, 23–25, 27–32, 34, 37, 39, 44, 45, 49, 50, 52], 2 studies used the Molecular Taxonomy of Breast Cancer International Consortium (METABRIC) [40, 42], 1 study used The Cancer Genome Atlas (TCGA) [41], 1 study used Haberman’s Cancer Survival Dataset [51], and 9 studies used hospital registry data [26, 33, 35, 36, 38, 43, 46–48]. The databases of 22 studies were public [2, 23–25, 27–32, 34, 37, 39–42, 44, 45, 49–52], and the databases of 9 studies were private [26, 33, 35, 36, 38, 43, 46–48]. The median sample size used for modeling was 37256 (range 200 to 659802) patients. Seven studies had a sample size of less than 1000 patients [26, 33, 36, 38, 41, 47, 51] (see S3 Table).

### Data preparation and modeling

In total, 31 studies conducted data preprocessing, among which 20 described missing value information and reported missing value processing strategies, including deleting directly, multiple imputation, and nearest neighbor algorithm [2, 25–29, 33, 36–40, 42–44, 46, 48–50, 52]. Eight studies detailed the feature selection process and reported the feature selection method, including a literature review and clinical availability, logistic regression, information gain ratio measurement, threshold-based preselection method and clustering, genetic algorithm, least absolute shrinkage and selectionator operator, and minimal redundancy maximal relevance [28, 29, 33, 40, 41, 43, 48, 49]. One study focused on the processing of outliers, and the algorithms used included the C-support vector classification filter, Adaboost, boosting, Adaboost SVM, and boosting SVM [26].

For the class imbalance, 24 studies showed class imbalance in the samples of the final model construction [2, 23, 24, 26, 28–37, 40–45, 47–50], and 7 of them dealt with this problem [28, 29, 34, 37, 42, 47, 49]. The methods included undersampling, bagging algorithm, SMOTE, PSO, K-means, KNN, and bagging. However, 2 studies used the method of randomly selecting the same number of samples from most classes as that from a few classes to balance the sample size of the two classes before modeling [39, 46], and 5 studies did not provide class imbalance data information [25, 27, 38, 51, 52].

For model presentation, 6 studies were presented as formulas [23, 29, 35, 36, 39, 44], 5 as graphs [24, 38, 47, 48, 52], and 16 as a combination of formulas and graphs [2, 25, 27, 30–32, 34, 40–43, 45, 46, 49–51]. Models were not presented in 4 studies [26, 28, 33, 37].

For the algorithms used in model construction, 5 studies used only one ML algorithm to build the model [25, 28, 33, 38, 50], and 26 studies used two or more ML algorithms and compared them [2, 23, 24, 26, 27, 29–32, 34–37, 39–49, 51, 52]. Common ML algorithms included DT (19 studies) [2, 23–26, 28, 29, 32, 34–38, 43, 46–48, 51, 52]; ANN(18 studies) [2, 23, 24, 26, 27, 30–33, 39, 42, 44, 46, 48–52]; SVM (16 studies) [30–32, 35–37, 39, 40, 42–48, 51]; LR (12 studies) [2, 24, 29, 34–36, 39, 40, 45, 48, 49, 52]; Bayesian classification (6 studies) [23, 24, 26, 27, 37, 47], KNN (3 studies) [34, 36, 39]; semisupervised learning (3 studies) [30–32]; ensemble learning including random forest, boosting, and random committee (10 studies) [26, 40–48]; and deep neural network (3 studies) [40, 41, 45] (see S4 Table).

### Information on model construction and performance evaluation

All 31 studies conducted internal validation, of which 27 used cross-validation [2, 23, 24, 26–32, 34–36, 38–47, 49–52], and 4 used random splitting [25, 33, 37, 38]. External validation was conducted in only one study [40], and model calibration was performed in only 1 study [48]. A total of 9 studies reported trying different hyperparameters on the model [31, 32, 34–36, 40, 43–45], but few studies reported details on hyperparameter tuning.

The common evaluation metrics of ML model classification and discrimination performance were as follows: 29 studies evaluated the accuracy of the model [2, 23–31, 33–42, 44–52], ranging from 0.510 to 0.971; 25 studies evaluated the sensitivity/recall [2, 23–25, 27–29, 31, 33, 34, 36–41, 43–46, 48–52], ranging from 0.037 to 1.000; 24 studies evaluated the specificity [2, 23, 25, 27–29, 31, 33, 34, 36–41, 43–46, 48–52], ranging from 0.008 to 0.993; 20 studies evaluated the AUC [26–34, 36, 39–43, 45, 47–49, 51], ranging from 0.500 to 0.972; 6 studies evaluated the precision/positive predictive value [23, 40, 41, 46–48], ranging from 0.549 to 1; 5 studies evaluated the F1 score [43, 45–47, 51], ranging from 0.369 to 0.966; 5 studies evaluated Mcc [40, 41, 46–48], ranging from 0 to 0.884; 2 studies evaluated the NPV [46, 47], ranging from 0 to 1; and 2 studies evaluated the G-mean [29, 34], ranging from 0.334 to 0.959.

In studies that compared of two or more algorithms, ANN had the best performance in 6 studies [27, 34, 39, 46, 49, 52], DT had the best performance in 4 studies [2, 23, 26, 34], the ensemble learning algorithm had the best performance in 4 studies [42, 43, 48, 51], semisupervised learning had the best performance in 3 studies [30–32], DNN had the best performance in 3 studies [40, 41, 45], SVM had the best performance in 2 studies [35, 37], LR had the best performance in 2 studies [24, 29], KNN had the best performance in 1 study [36], and Naive Bayes had the best performance in 1 study [47] (see S5 Table).

### Candidate predictors

The median number of candidate predictors used was 16 (range: 3~625); 29 studies used only clinical data [2, 23–39, 42–52], 1 study combined clinical data with molecular data for prediction [40], and 1 study combined clinical data, molecular data and pathological image data for prediction [41]. We ranked the frequency of the use of certain predictors from high to low. The commonly used candidate predictors included age, stage of cancer, grade, tumor size, race, marital status, number of nodes, histology, number of positive nodes, primary site code, extension of tumor, behavior/behavior code, lymph node involvement, site-specific surgery code, number of primaries, radiation, received radiation, estrogen receptor (ER) status, and progesterone receptor (PR) status (see Table 4).

**Table 4:** Rank of the candidate predictors used in 31 studies.

Rank	Candidate predictor	Description	Number (n)	Percentage (%)
**1**	Age	Age at diagnosis	26	83.9
**2**	Stage of cancer	Defined by size of cancer tumor and its spread	23	74.2
**3**	Grade	Appearance of tumors and their differentiability	22	71.0
**4**	Tumor size	Diameter of tumor	21	67.7
**5**	Race	Recoded race of the patient. Ethnicity: White, Black, Chinese, etc.	19	61.3
**6**	Marital status	Patient’s marital status at the time of diagnosis: Married, single, divorced, widowed, separated	19	61.3
**7**	Number of nodes	Total nodes (positive/negative) examined	19	61.3
**8**	Histology	The microscopic composition of cells and/or tissue for a specific primary	18	58.1
**9**	Number of positive nodes	When lymph nodes are involved in cancer, they are called positive	18	58.1
**10**	Primary site code	Presence of tumor at particular location in body. Topographical classification of cancer	17	54.8
**11**	Extension of tumor	Defines spread of tumor relative to breast	17	54.8
**12**	Behavior/behavior code	In situ or malignant	15	48.4
**13**	Lymph node involvement	None, minimal, significant, etc.	14	45.2
**14**	Site-specific surgery code	Information on surgery during first course of therapy, whether cancer-directed or not	13	41.9
**15**	Number of primaries	Number of primary tumors	13	41.9
**16**	Radiation	None, beam radiation, radioisotopes, refused, recommended, etc.	11	35.5
**17**	Received radiation	Whether the patient had been treated with radiotherapy or not	8	25.8
**18**	Estrogen receptor (ER) status	Breast cancers with this hormone receptor are called “ER positive”	7	22.6
**19**	Progesterone receptor (PR) status	Breast cancers with this hormone receptor are called “PR positive”	7	22.6

Fifteen studies ranked the degree to which the predictors contributed to the outcome [2, 23, 25, 27, 32, 33, 37, 38, 42, 43, 46, 48–50, 52]. Four studies reported sequencing methods, including sensitivity analysis in networks [2, 27], DT information gain measurement [23, 25], sensitivity scores in rules [38], and correlation coefficients [33] (see S6 Table).

## Discussion

To the best of our knowledge, this is the first systematic review of the application of ML to breast cancer survival prediction, and accurate 5-year survival predictions are very important for further research. After a systematic analysis of 31 studies, we found that there is a need for the standardization and validation of the different algorithms of models for predicting breast cancer survival and for the exploration of the significance of applying the predictive model to clinical practice.

Most studies based on authoritative databases use standardized and open-access tumor information that is updated regularly, but the question of whether a model using public databases could be used locally should be considered. In addition, some public databases that were established earlier are problematic because clinical practices change over time, and the use of historical data that are too old or a data collection time period that is too long to develop the model will result in the loss of clinical significance [53]. Therefore, researchers should consider focusing more on data management to improve the speed of building models and consider establishing online real-time prediction models. A small number of studies are based on local hospital registration data, but private data require informed consent and ethics committee approval before sharing as well as proper processing (such as anonymity completely). Therefore, the use of private data prevents other scholars from verifying the results of the model and comparing different models.

The number of samples included in this study is uneven. The minimum sample size is 200 patients, and 7 model samples include less than 1000 patients. ML algorithms are often applied to the processing of multidimensional data, and the default application condition is large sample data [54, 55]. The use of too little data in the training model will often lead to overfitting of the model and reduce the generalization ability. In addition, medical data typically contain a large amount of data, outliers, noise redundancy, imbalance, deletion and irrelevant variables [56]. The original dataset will thus cause poor performance of the subsequent prediction model and will become a bottleneck in the process of data mining. Therefore, the process of data preprocessing, including data reduction, data cleaning, data transformation and data integration, is crucial [57] and typically comprises 70~80% of the workload of data mining [58]. However, many of the studies included in this systematic review did not take these key steps. High-quality models depend on high-quality data. In future studies, researchers should not only select appropriate algorithms and perform performance comparisons but also focus on exploring methods for data cleaning and pretreatment and improving the quality and quantity of the modeling data.

Initially, researchers used traditional ML for model construction and then gradually combined and optimized multiple learning models with weak performance to produce ensemble learning algorithms, which have high prediction accuracy and strong generalization ability [59, 60]. However, the above two algorithms are shallow learning algorithms. Although these algorithms play a role, they are often unable to effectively complete tasks such as high-dimensional data processing and large computations when faced with massive data. Therefore, driven by the background of big data cloud computing, deep learning algorithms have been proposed and have gradually become hotspots in breast cancer prediction research. These algorithms are better able to analyze data and model the complex relationship between prognostic variables. The algorithms include factors that depend on time as well as those that interact with other factors associated with prognosis in a nonlinear manner.

In complex modeling problems, there is generally no single algorithm that fits all problems. Different techniques can be combined to produce the best performance, so researchers must compare different ML algorithms or ML algorithms with traditional modeling algorithms. The most commonly used algorithms in this systematic review are ANN, DT, SVM, LR and ensemble learning. Among them, the performance of ANN and DT is better. However, overall, compared with LR/Cox regression model, the performance of the ML algorithm does not necessarily improve, similar to the results of previous studies [61–63].

Model validation is divided into internal and external validation, and internal validation is performed using the dataset randomly obtained from the original dataset, which can be completed by dividing the sample validation. In this study, most of the included studies used cross-validation and random splitting for internal validation, which makes it difficult to avoid overfitting, thus limiting the accuracy of the validation results [64]. External validation requires the development of the queue based on the independence and validation of samples, which is the gold standard of model performance [65, 66]. We found that only 1 study performed external verification of the model. The lack of external validation in multicenter studies with large samples prevents one from determining whether a model is applicable in different scenarios, which can prevent the use of the model, as well as its stability and universality. Thus, data extrapolation should be performed with caution. The lack of practical application of the model in clinical practice may affect the ability of clinicians to make treatment decisions and estimate prognosis. Calibration compares the observed probability and predicted probability of the occurrence of results, which is the key to model development [67]. Only 1 study performed model calibration, and the actual availability of uncalibrated models is limited [68]. Therefore, it is recommended that researchers consider this step and report modeling information in detail.

Compared with the traditional statistical model, the ML algorithm has the black box property. The interpretation and understanding of the model is a key problem [69]. Researchers have difficulties in knowing what happened in the process of prediction and the resulting process, i.e., which variables had the greatest influence on survival and which subgroups of patients showed similar results. Answering these questions can help doctors choose the appropriate treatment and can also eliminate the non-important factors of breast cancer to reduce the time and cost of data collection and treatment. However, whether this problem exists in most models, especially deep learning models, is unknown [70, 71]. The development of this problem and the complex function of internal work are not easy to explain, leading to inappropriate evaluation and feedback to improve the output. In contrast, DT models have excellent interpretability, but their performance still needs to be further optimized [51, 72]. Therefore, compared with focusing only on prediction performance, further understanding of the underlying dynamics of the algorithm has become a research hotspot and led to an increasing number of studies being performed [69].

Regarding factors influencing breast cancer prognosis, screening appropriate predictors as independent variables is an important step in model construction. In previous studies, predictors mostly included patients’ demographic characteristics, medical history, treatment information, and the clinicopathological characteristics of tumors at different disease stages. In this systematic review, we summarize the most commonly used predictors similar to the results of previous studies [4].

Age, disease stage, grade, tumor size, race, marital status, number of nodes, histology, number of positive nodes and primary site code have been entered into many predictive models as predictors, given that these factors represent key risk factors for onset and survival in breast cancer. These variables were also used in studies on decision-making analysis in relation to breast cancer [73–75]. In the future, the possible mechanisms underlying the occurrence and development of breast cancer could be further studied from these perspectives, which also suggests that more suitable predictors for clinical practice can be identified. The ML predictive models applied in this systematic review can be translated into tools for clinical treatment decision-making. Visualization of some of the outcomes will be implemented in the research database and used by the clinicians at the hospital to analyze the survival of breast cancer patients.

With the development of molecular biology, some molecular indicators, such as gene expression and mutation, have also become predictors. Compared with a single data-driven prediction model, in recent years, researchers have incorporated multiple types of data into prediction. The rapid increase in the number of features from different data sources and the use of heterogeneous features have led to great challenges in survival prediction. With the deepening of research on breast cancer, many new variables that are significantly related to breast cancer prognosis have been gradually discovered [14, 76, 77], such as the level of anxiety and depression. Thus, the above factors should be taken into account in the prediction. This notion illustrates the true complexity of breast cancer as a disease, highlights the importance of the mechanisms involved, and highlights some of the confusion among researchers in selecting the most appropriate prediction model.

The number of candidate predictors and the correlation between them will affect model performance. Therefore, feature selection becomes particularly important. Feature selection identifies the most important variables in the dataset while maintaining classification quality. A reduction in the number of predictors and the burden of data collection can reduce the fitting and complexity of the model and help researchers interpret and understand the model. However, many studies did not report the feature selection process, which may be related to the ability of some classification models to deal with high-dimensional datasets (e.g., RF, SVM, DT), or the features included in the models may have been selected based on prior research or clinical importance [14, 78].

No quality assessment criteria have been established specifically for systematic reviews of ML research. Existing guidelines, such as CHARMS [21] and TRIPOD [79], do not consider the characteristics and related biases of ML models. There have been studies using improved quality assessment criteria to adapt to ML system evaluation [62, 80, 81], but they have not been widely accepted. Therefore, with the increasing application of ML in prediction and other fields, it is recommended that guidelines be developed for reporting and evaluating ML prediction model research in the medical field and to serve as a standard for publication to improve the quality of related papers.

## Limitations

This study has some limitations. First, only English studies were included, so publication bias may be present. Second, the excessive differences of the included studies limit the comparison between studies and prohibits the use of meta-analysis [82, 83]. Finally, most of the included studies did not report the key steps in model development and validation. In addition, the information on predictive performance (such as true positive, false positive, true negative, and false negative in the confusion matrix) was insufficient, and most of the studies only described a single dimension of predictive performance. Therefore, it is recommended that comprehensive methodological information, such as missing value processing, outlier value processing, class imbalance processing, hyperparameter tuning, feature selection variable importance ranking processing, model evaluation and validation, be reported in detail along with the model performance, including detailed information on the suitability and acceptability of classification, discrimination and calibration measures.

## Conclusion

ML has become a new methodology for breast cancer survival prediction, and there is still much room for improvement and potential for further model construction. The existing prediction models still face limitations related to a lack of data preprocessing steps, the excessive differences of sample feature selection, and issues related to validation and promotion. The model performance still needs to be further optimized, and other barriers should be addressed. Researchers and medical workers should connect with reality, choose a model carefully, use the model in clinical practice after verification, and use rigorous design and validation methods with a large sample of high-quality research data on the basis of previous findings. The applicability and limitation of these models should be evaluated strictly to improve the degree of accuracy for breast cancer survival prediction.

## Supporting information

S1 Table PRISMA checklist. (DOC)

S2 Table Search strategy and results. (DOCX)

S3 Table Primary information of the 31 studies. (DOCX)

S4 Table Data preparation and modeling process of the 31 studies. (DOCX)

S5 Table Model construction and performance evaluation of the 31 studies. (DOCX)

S6 Table Candidate predictors used in the 31 studies. (DOCX)

## Abbreviations

USA: United States of America
SEER: Surveillance Epidemiology and End Result
AUC: area calculated under the receiver operating characteristic (ROC) curve
ANN: artificial neural network
DT: decision trees
LR: logistic regression
Mcc: Matthew’s correlation coefficient
NPV: negative predictive value
FPR: false positive rate
FDR: false discovery rate
FNR: False Negative Rate
MLP: multi-layer perceptron
KNN: K-nearest neighbors
SVM: support vector machines
RF: random forest
DNN: deep neural network
SMOTE: synthetic minority oversampling technique
PSO: particle swarm optimization

## References

[1] Bray F, Ferlay J, Soerjomataram I, Siegel R, Torre L, Jemal A. Global cancer statistics 2018: GLOBOCAN estimates of incidence and mortality worldwide for 36 cancers in 185 countries. CA: a cancer journal for clinicians. 2018. 10.3322/caac.21492 30207593

[2] Delen D, Walker G, Kadam A. Predicting breast cancer survivability: a comparison of three data mining methods. Artificial intelligence in medicine. 2005;34(2):113–27. 10.1016/j.artmed.2004.07.002 15894176

[3] Polyak K. Heterogeneity in breast cancer. The Journal of clinical investigation. 2011;121(10):3786–8. 10.1172/JCI60534 21965334 PMC3195489

[4] Altman, Douglas G. Prognostic models: a methodological framework and review of models for breast cancer. Cancer Investigation. 2009;27(3):235–43. 10.1080/07357900802572110 19291527

[5] Clark GM. Do we really need prognostic factors for breast cancer? Breast cancer research and treatment. 1994;30(2):117–26. 10.1007/BF00666054 7949209

[6] Altman DG, Royston P. What do we mean by validating a prognostic model? Statistics in Medicine. 2015;19(4):453–73. 10.1002/(sici)1097-0258(20000229)19:4<453::aid-sim350>3.0.co;2-5 10694730

[7] Stone P, Lund S. Predicting prognosis in patients with advanced cancer. Annals of Oncology Official Journal of the European Society for Medical Oncology. 2007;18(6):971. 10.1093/annonc/mdl343 17043092

[8] Kourou K, Exarchos TP, Exarchos KP, Karamouzis MV, Fotiadis DI. Machine learning applications in cancer prognosis and prediction. Computational and structural biotechnology journal. 2015;13:8–17. 10.1016/j.csbj.2014.11.005 25750696 PMC4348437

[9] Obermeyer Z, Emanuel EJ. Predicting the Future—Big Data, Machine Learning, and Clinical Medicine. The New England journal of medicine. 2016;375(13):1216–9. 10.1056/NEJMp1606181 27682033 PMC5070532

[10] Acs B, Rantalainen M, Hartman J. Artificial intelligence as the next step towards precision pathology. Journal of internal medicine. 2020;288(1):62–81. 10.1111/joim.13030 32128929

[11] Yassin NIR, Omran S, El Houby EMF, Allam H. Machine learning techniques for breast cancer computer aided diagnosis using different image modalities: A systematic review. Computer methods and programs in biomedicine. 2018;156:25–45. 10.1016/j.cmpb.2017.12.012 29428074

[12] Crowley RJ, Tan YJ, Ioannidis JPA. Empirical assessment of bias in machine learning diagnostic test accuracy studies. Journal of the American Medical Informatics Association: JAMIA. 2020;27(7):1092–101. 10.1093/jamia/ocaa075 32548642 PMC7647361

[13] Gardezi SJS, Elazab A, Lei B, Wang T. Breast Cancer Detection and Diagnosis Using Mammographic Data: Systematic Review. Journal of medical Internet research. 2019;21(7):e14464. 10.2196/14464 31350843 PMC6688437

[14] Richter AN, Khoshgoftaar TM. A review of statistical and machine learning methods for modeling cancer risk using structured clinical data. Artificial intelligence in medicine. 2018;90:1–14. 10.1016/j.artmed.2018.06.002 30017512

[15] Izci H, Tambuyzer T, Tuand K, Depoorter V, Laenen A, Wildiers H, et al. A Systematic Review of Estimating Breast Cancer Recurrence at the Population Level With Administrative Data. Journal of the National Cancer Institute. 2020;112(10):979–88. 10.1093/jnci/djaa050 32259259 PMC7566328

[16] Juwara L, Arora N, Gornitsky M, Saha-Chaudhuri P, Velly AM. Identifying predictive factors for neuropathic pain after breast cancer surgery using machine learning. International journal of medical informatics. 2020;141:104170. 10.1016/j.ijmedinf.2020.104170 32544823

[17] Yang L, Fu B, Li Y, Liu Y, Huang W, Feng S, et al. Prediction model of the response to neoadjuvant chemotherapy in breast cancers by a Naive Bayes algorithm. Computer methods and programs in biomedicine. 2020;192:105458. 10.1016/j.cmpb.2020.105458 32302875

[18] Sutton EJ, Onishi N, Fehr DA, Dashevsky BZ, Sadinski M, Pinker K, et al. A machine learning model that classifies breast cancer pathologic complete response on MRI post-neoadjuvant chemotherapy. Breast cancer research: BCR. 2020;22(1):57. 10.1186/s13058-020-01291-w 32466777 PMC7254668

[19] Takada M, Sugimoto M, Masuda N, Iwata H, Kuroi K, Yamashiro H, et al. Prediction of postoperative disease-free survival and brain metastasis for HER2-positive breast cancer patients treated with neoadjuvant chemotherapy plus trastuzumab using a machine learning algorithm. Breast cancer research and treatment. 2018;172(3):611–8. 10.1007/s10549-018-4958-9 30194511

[20] Phung MT, Tin Tin S, Elwood JM. Prognostic models for breast cancer: a systematic review. BMC cancer. 2019;19(1):230. 10.1186/s12885-019-5442-6 30871490 PMC6419427

[21] Moons KG, de Groot JA, Bouwmeester W, Vergouwe Y, Mallett S, Altman DG, et al. Critical appraisal and data extraction for systematic reviews of prediction modelling studies: the CHARMS checklist. PLoS medicine. 2014;11(10):e1001744. 10.1371/journal.pmed.1001744 25314315 PMC4196729

[22] Moons KGM, Wolff RF, Riley RD, Whiting PF, Westwood M, Collins GS, et al. PROBAST: A Tool to Assess Risk of Bias and Applicability of Prediction Model Studies: Explanation and Elaboration. Annals of internal medicine. 2019;170(1):W1–w33. 10.7326/M18-1377 30596876

[23] Bellaachia A, E G. Predicting Breast Cancer Survivability Using Data Mining Techniques2006:[1–4 pp.]. Available from: https://vpns.jlu.edu.cn/http/77726476706e69737468656265737421e3e40f862f3972587b06c7af9758/detail_38502727e7500f262131a1f059e6d921db72c3d5948903771921b0a3ea255101e580949000984f4b5e87a378de3a694b55e004485bcadaf0a5d1d4ce4b994fb7beb34a549f1df8f78e7931e8537ccd77?

[24] Endo A, Shibata T, H T. Comparison of Seven Algorithms to Predict Breast Cancer Survival. International Journal of Biomedical Soft Computing and Human Sciences: the official journal of the Biomedical Fuzzy Systems Association. 2008;13(2):11–6.

[25] Khan MU, Choi JP, Shin H, Kim M. Predicting breast cancer survivability using fuzzy decision trees for personalized healthcare. Annual International Conference of the IEEE Engineering in Medicine and Biology Society IEEE Engineering in Medicine and Biology Society Annual International Conference. 2008;2008:5148–51. 10.1109/IEMBS.2008.4650373 19163876

[26] Thongkam J, Xu G, Zhang Y, Huang F, editors. Support Vector Machine for Outlier Detection in Breast Cancer Survivability Prediction. Advanced Web & Networktechnologies, & Applications; 2008.

[27] Choi J, Han T, Park P. A Hybrid Bayesian Network Model for Predicting BreastCancer Prognosis. J Kor Soc Med Informatics. 2009;15(1):49–57.

[28] Liu YQ, Wang C, Zhang L, editors. Decision Tree Based Predictive Models for Breast Cancer Survivability on Imbalanced Data. International Conference on Bioinformatics & Biomedical Engineering; 2009.

[29] Wang KJ, Makond B, Wang KM. An improved survivability prognosis of breast cancer by using sampling and feature selection technique to solve imbalanced patient classification data. BMC Med Inform Decis Mak. 2013;13:124. 10.1186/1472-6947-13-124 24207108 PMC3829096

[30] Kim J, Shin H. Breast cancer survivability prediction using labeled, unlabeled, and pseudo-labeled patient data. Journal of the American Medical Informatics Association: JAMIA. 2013;20(4):613–8. 10.1136/amiajnl-2012-001570 23467471 PMC3721173

[31] Park K, Ali A, Kim D, An Y, Kim M, Shin H. Robust predictive model for evaluating breast cancer survivability. Engineering Applications of Artificial Intelligence. 2013;26(9):2194–205.

[32] Shin H, Nam Y. A coupling approach of a predictor and a descriptor for breast cancer prognosis. BMC Med Genomics. 2014;7 Suppl 1:S4. 10.1186/1755-8794-7-S1-S4 25080202 PMC4101306

[33] Wang TN, Cheng CH, Chiu HW. Predicting post-treatment survivability of patients with breast cancer using Artificial Neural Network methods. Conf Proc IEEE Eng Med Biol Soc. 2015;2013:1290–3. 10.1109/EMBC.2013.6609744 24109931

[34] Wang KJ, Makond B, Chen KH, Wang KM. A hybrid classifier combining SMOTE with PSO to estimate 5-year survivability of breast cancer patients. Applied Soft Computing. 2014;20:15–24.

[35] Chao CM, Yu YW, Cheng BW, Kuo YL. Construction the model on the breast cancer survival analysis use support vector machine, logistic regression and decision tree. J Med Syst. 2014;38(10):106. 10.1007/s10916-014-0106-1 25119239

[36] Garcia-Laencina PJ, Abreu PH, Abreu MH, Afonoso N. Missing data imputation on the 5-year survival prediction of breast cancer patients with unknown discrete values. Computers in biology and medicine. 2015;59:125–33. 10.1016/j.compbiomed.2015.02.006 25725446

[37] Lotfnezhad Afshar H, Ahmadi M, Roudbari M, Sadoughi F. Prediction of breast cancer survival through knowledge discovery in databases. Glob J Health Sci. 2015;7(4):392–8. 10.5539/gjhs.v7n4p392 25946945 PMC4802184

[38] Khalkhali HR, Afshar HL, Esnaashari O, Jabbari N. Applying Data Mining Techniques to Extract Hidden Patterns about Breast Cancer Survival in an Iranian Cohort Study. Journal of Research in Health Sciences. 2016;16(1):31. 27061994 PMC7189091

[39] Shawky DM, Seddik AF. On the Temporal Effects of Features on the Prediction of Breast Cancer Survivability. Current Bioinformatics. 2017;12(4).

[40] Sun D, Wang M, Li A. A multimodal deep neural network for human breast cancer prognosis prediction by integrating multi-dimensional data. IEEE/ACM Trans Comput Biol Bioinform. 2018. 10.1109/TCBB.2018.2806438 29994639

[41] Sun D, Li A, Tang B, Wang M. Integrating genomic data and pathological images to effectively predict breast cancer clinical outcome. Computer methods and programs in biomedicine. 2018;161:45–53. 10.1016/j.cmpb.2018.04.008 29852967

[42] Zhao M, Tang Y, Kim H, Hasegawa K. Machine Learning With K-Means Dimensional Reduction for Predicting Survival Outcomes in Patients With Breast Cancer. Cancer Inform. 2018;17:1176935118810215. 10.1177/1176935118810215 30455569 PMC6238199

[43] Fu B, Liu P, Lin J, Deng L, Hu K, Zheng H. Predicting Invasive Disease-Free Survival for Early-stage Breast Cancer Patients Using Follow-up Clinical Data. IEEE Trans Biomed Eng. 2018. 10.1109/TBME.2018.2882867 30475709

[44] Lu H, Wang H, Yoon SW. A dynamic gradient boosting machine using genetic optimizer for practical breast cancer prognosis. Expert Systems with Applications. 2019;116:340–50.

[45] Abdikenov B, Iklassov Z, Sharipov A, Hussain S, Jamwal PK. Analytics of Heterogeneous Breast Cancer Data Using Neuroevolution. IEEE Access. 2019;7:18050–60.

[46] Kalafi EY, Nor NAM, Taib NA, Ganggayah MD, Town C, Dhillon SK. Machine Learning and Deep Learning Approaches in Breast Cancer Survival Prediction Using Clinical Data. Folia biologica. 2019;65(5–6):212–20. 32362304 10.14712/fb2019065050212

[47] Shouket T, Mahmood S, Hassan MT, Iftikhar A, editors. Overall and Disease-Free Survival Prediction of Postoperative Breast Cancer Patients using Machine Learning Techniques. 2019 22nd International Multitopic Conference (INMIC); 2019.

[48] Ganggayah MD, Taib NA, Har YC, Lio P, Dhillon SK. Predicting factors for survival of breast cancer patients using machine learning techniques. BMC Med Inform Decis Mak. 2019;19(1):48. 10.1186/s12911-019-0801-4 30902088 PMC6431077

[49] Simsek S, Kursuncu U, Kibis E, AnisAbdellatif M, Dag A. A hybrid data mining approach for identifying the temporal effects of variables associated with breast cancer survival. Expert Systems with Applications. 2020;139.

[50] Salehi M, Lotfi S, Razmara J. A Novel Data Mining on Breast Cancer Survivability Using MLP Ensemble Learners. The Computer Journal. 2020;63(3):435–47.

[51] Tang C, Ji J, Tang Y, Gao S, Tang Z, Todo Y. A novel machine learning technique for computer-aided diagnosis. Engineering Applications of Artificial Intelligence. 2020;92.

[52] Hussain OI. Predicting Breast Cancer Survivability A Comparison of Three Data Mining Methods. Cihan University-Erbil Journal of Humanities and Social Sciences. 2020;14(1):17–30.

[53] Hickey GL, Grant SW, Murphy GJ, Bhabra M, Pagano D, McAllister K, et al. Dynamic trends in cardiac surgery: why the logistic EuroSCORE is no longer suitable for contemporary cardiac surgery and implications for future risk models. European journal of cardio-thoracic surgery: official journal of the European Association for Cardio-thoracic Surgery. 2013;43(6):1146–52. 10.1093/ejcts/ezs584 23152436 PMC3655624

[54] Aj A, Pdy B, Sm C, Gkka D, Kt A. Efficient Machine Learning for Big Data: A Review. Big Data Research. 2015;2(3):87–93.

[55] van der Ploeg T, Austin PC, Steyerberg EW. Modern modelling techniques are data hungry: a simulation study for predicting dichotomous endpoints. BMC medical research methodology. 2014;14:137. 10.1186/1471-2288-14-137 25532820 PMC4289553

[56] Razzaghi T, Roderick O, Safro I, Marko N. Multilevel Weighted Support Vector Machine for Classification on Healthcare Data with Missing Values. PloS one. 2016;11(5):e0155119. 10.1371/journal.pone.0155119 27195952 PMC4873242

[57] Han J, Micheline K. Data Mining: Concepts and Techniques. Data Mining Concepts Models Methods Algorithms Second Edition. 2006;5(4):1–18.

[58] Pérez J, Iturbide E, Olivares V, Hidalgo M, Martínez A, Almanza N. A Data Preparation Methodology in Data Mining Applied to Mortality Population Databases. J Med Syst. 2015;39(11):152. 10.1007/s10916-015-0312-5 26385549 PMC4575356

[59] Khamparia A, Singh A, Anand D, Gupta D, Khanna A, Arun Kumar N, et al. A novel deep learning-based multi-model ensemble method for the prediction of neuromuscular disorders. Neural Computing Applications. 2018.

[60] Ko HR, Sabourin R, Britt A, editors. Combining Diversity and Classification Accuracy for Ensemble Selection in Random Subspaces. Neural Networks, 2006 IJCNN ’06 International Joint Conference on; 2006.

[61] Gang L. A review of automatic selection methods for machine learning algorithms and hyper-parameter values. Network Modeling Analysis in Health Informatics & Bioinformatics. 2016;5(1):18.

[62] Senanayake S, White N, Graves N, Healy H, Baboolal K, Kularatna S. Machine learning in predicting graft failure following kidney transplantation: A systematic review of published predictive models. International journal of medical informatics. 2019;130:103957. 10.1016/j.ijmedinf.2019.103957 31472443

[63] Christodoulou E, Ma J, Collins GS, Steyerberg EW, Verbakel JY, Van Calster B. A systematic review shows no performance benefit of machine learning over logistic regression for clinical prediction models. Journal of clinical epidemiology. 2019;110:12–22. 10.1016/j.jclinepi.2019.02.004 30763612

[64] Collins GS, de Groot JA, Dutton S, Omar O, Shanyinde M, Tajar A, et al. External validation of multivariable prediction models: a systematic review of methodological conduct and reporting. BMC medical research methodology. 2014;14:40. 10.1186/1471-2288-14-40 24645774 PMC3999945

[65] Laupacis A, Sekar N, Stiell IG. Clinical prediction rules. A review and suggested modifications of methodological standards. Jama. 1997;277(6):488–94. 9020274

[66] Vergouwe Y, Moons KG, Steyerberg EW. External validity of risk models: Use of benchmark values to disentangle a case-mix effect from incorrect coefficients. American journal of epidemiology. 2010;172(8):971–80. 10.1093/aje/kwq223 20807737 PMC2984249

[67] Steyerberg EW. Clinical Prediction Models. Springer US. 2009; 10.1007/978-0-387-77244-8

[68] Steyerberg EW, Vickers AJ, Cook NR, Gerds T, Gonen M, Obuchowski N, et al. Assessing the Performance of Prediction Models. Epidemiology. 2010;21(1):128–38. 10.1097/EDE.0b013e3181c30fb2 20010215 PMC3575184

[69] Riccardo G, Anna M, Salvatore R, Franco T, Fosca G, Dino P. A Survey Of Methods For Explaining Black Box Models. ACM Computing Surveys. 2018;51(5):1–42.

[70] Dembrower K, Liu Y, Azizpour H, Eklund M, Strand F. Comparison of a Deep Learning Risk Score and Standard Mammographic Density Score for Breast Cancer Risk Prediction. Radiology. 2019;294(2):190872. 10.1148/radiol.2019190872 31845842

[71] Wang H, Li Y, Khan SA, Luo Y. Prediction of breast cancer distant recurrence using natural language processing and knowledge-guided convolutional neural network. Artificial intelligence in medicine. 2020;110:101977. 10.1016/j.artmed.2020.101977 33250149 PMC7983067

[72] Lior R, Oded M. Data Mining with Decision Trees: Theory and Applications: WORLD SCIENTIFIC; 2007.

[73] Ibrahim N, Kudus A, Daud I, Abu Bakar M. Decision Tree for Competing Risks Survival Probability in Breast Cancer Study. Proc Wrld Acad Sci Eng Tech. 2008.

[74] Cianfrocca M, Goldstein LJ. Prognostic and predictive factors in early-stage breast cancer. The oncologist. 2004;9(6):606–16. 10.1634/theoncologist.9-6-606 15561805

[75] Kurt TI. Using Kaplan–Meier analysis together with decision tree methods (C&RT, CHAID, QUEST, C4.5 and ID3) in determining recurrence-free survival of breast cancer patients. Expert Systems with Applications. 2009.

[76] Wang X, Wang N, Zhong L, Wang S, Zheng Y, Yang B, et al. Prognostic value of depression and anxiety on breast cancer recurrence and mortality: a systematic review and meta-analysis of 282,203 patients. Molecular psychiatry. 2020;25(12):3186–97. 10.1038/s41380-020-00865-6 32820237 PMC7714689

[77] Escala-Garcia M, Morra A, Canisius S, Chang-Claude J, Kar S, Zheng W, et al. Breast cancer risk factors and their effects on survival: a Mendelian randomisation study. BMC medicine. 2020;18(1):327. 10.1186/s12916-020-01797-2 33198768 PMC7670589

[78] Walsh C, Hripcsak G. The effects of data sources, cohort selection, and outcome definition on a predictive model of risk of thirty-day hospital readmissions. Journal of biomedical informatics. 2014;52:418–26. 10.1016/j.jbi.2014.08.006 25182868 PMC4261028

[79] Collins GS, Reitsma JB, Altman DG, Moons KGM. Transparent Reporting of a Multivariable Prediction Model for Individual Prognosis or Diagnosis (TRIPOD): The TRIPOD Statement. European Urology. 2015;67(6):1142–51. 10.1016/j.eururo.2014.11.025 25572824

[80] Qiao N. A systematic review on machine learning in sellar region diseases: quality and reporting items. Endocrine connections. 2019;8(7):952–60. 10.1530/EC-19-0156 31234143 PMC6612064

[81] Silva K, Lee WK, Forbes A, Demmer RT, Barton C, Enticott J. Use and performance of machine learning models for type 2 diabetes prediction in community settings: A systematic review and meta-analysis. International journal of medical informatics. 2020;143:104268. 10.1016/j.ijmedinf.2020.104268 32950874

[82] Thompson SG. Why sources of heterogeneity in meta-analysis should be investigated. Bmj. 1994;309(6965):1351–5. 10.1136/bmj.309.6965.1351 7866085 PMC2541868

[83] Blettner M, Sauerbrei W, Schlehofer B, Scheuchenpflug T, Friedenreich C. Traditional reviews, meta-analyses and pooled analyses in epidemiology. International journal of epidemiology. 1999;28(1):1–9. 10.1093/ije/28.1.1 10195657

