# Supplementary material for: Predicting breast cancer 5-year survival using machine learning: A systematic review
Source: PLoS One. 2021 Apr 16;16(4):e0250370. doi: 10.1371/journal.pone.0250370 (PMC8051758; doi:10.1371/journal.pone.0250370)
Supplement: S2 Table — (DOCX) [file pone.0250370.s002.docx]

**S2 Table.** **Search strategy and results.**

| **Database: Web of Science Core Collection** | | |
| --- | --- | --- |
| #1 | ALL FIELDS: (Breast Neoplasms) OR ALL FIELDS: (breast cancer) OR ALL FIELDS: (breast tumour) OR ALL FIELDS: (breast neoplasm) OR ALL FIELDS: (breast carcinoma) Indexes=SCI-EXPANDED, SSCI, A&HCI, CPCI-S, ESCI, CCR-EXPANDED, IC Timespan=All years | 558,223 |
| #2 | ALL FIELDS: ('Linear Discriminant Analysis') OR ALL FIELDS: ('K nearest neighbor') OR ALL FIELDS: ('K nearest neighbors') OR ALL FIELDS: ('K nearest neighbour') OR ALL FIELDS: ('K nearest neighbours') OR ALL FIELDS: ('K means') OR ALL FIELDS: ('Decision Tree') OR ALL FIELDS: ('Decision Trees') OR ALL FIELDS: ('Random Forest') OR ALL FIELDS: ('Random Forests') OR ALL FIELDS: ('Naive Bayes') OR ALL FIELDS: ('Naive Bayesian') OR ALL FIELDS: ('Support Vector Machine') OR ALL FIELDS: ('Support Vector Machines') OR ALL FIELDS: ('Deep Learning') OR ALL FIELDS: ('Neural Network') OR ALL FIELDS: ('AdaBoost') OR ALL FIELDS: ('XGBoost') OR ALL FIELDS: ('Gradient Boosting') OR ALL FIELDS: ('Algorithm') OR ALL FIELDS: ('Machine Learning') OR ALL FIELDS: ('Supervised Learning') OR ALL FIELDS: ('Unsupervised Learning') OR ALL FIELDS: ('Supervised Machine Learning') OR ALL FIELDS: ('Unsupervised Machine Learning') Indexes=SCI-EXPANDED, SSCI, A&HCI, CPCI-S, ESCI, CCR-EXPANDED, IC Timespan=All years | 2,876,678 |
| #3 | ALL FIELDS:(surviv*) Indexes=SCI-EXPANDED, SSCI, A&HCI, CPCI-S, ESCI, CCR-EXPANDED, IC Timespan=All years | 1,359,818 |
| #4 | #1 AND #2 AND #3 | 3,994 |
|  | | |
| **Database: PUBMED(include MEDLINE)** | | |
| #1 | ((((breast cancer) OR (breast tumour)) OR (breast neoplasm)) OR (breast carcinoma)) OR ("Breast Neoplasms"[Mesh]) | 427,826 |
| #2 | (((((((((((((((((((((((((‘Linear Discriminant Analysis’) OR (‘K nearest neighbor’)) OR (‘K nearest neighbors’)) OR (‘K nearest neighbour’)) OR (‘K nearest neighbours’)) OR (‘K means’)) OR (‘Decision Tree’)) OR (‘Decision Trees’)) OR (‘Random Forest’)) OR (‘Random Forests’)) OR (‘Naive Bayes’)) OR (‘Naive Bayesian’)) OR (‘Support Vector Machine’)) OR (‘Support Vector Machines’)) OR (‘Deep Learning’)) OR (‘Neural Network’)) OR (‘AdaBoost’)) OR (‘XGBoost’)) OR (‘Gradient Boosting’)) OR (‘Algorithm’)) OR (‘Machine Learning’)) OR (‘Supervised Learning’)) OR (‘Unsupervised Learning’)) OR (‘Supervised Machine Learning’)) OR (‘Unsupervised Machine Learning’)) OR (‘Machine Learning’[Mesh]) | 655,945 |
| #3 | surviv* | 1,474,684 |
| #4 | #1 AND #2 AND #3 | 1,521 |

| **Database: Embase** | | |
| --- | --- | --- |
| #1 | breast AND cancer OR (breast AND tumour) OR (breast AND neoplasm) OR (breast AND carcinoma) OR 'breast tumor'/exp | 685,488 |
| #2 | 'linear discriminant analysis' OR 'k nearest neighbor' OR 'k nearest neighbors' OR 'k nearest neighbour' OR 'k nearest neighbours' OR 'k means' OR 'decision tree' OR 'decision trees' OR 'random forest' OR 'random forests' OR 'naive bayes' OR 'naive bayesian' OR 'support vector machine' OR 'support vector machines' OR 'deep learning' OR 'neural network' OR 'adaboost' OR 'xgboost' OR 'gradient boosting' OR 'algorithm' OR 'machine learning' OR 'supervised learning' OR 'unsupervised learning' OR 'supervised machine learning' OR 'unsupervised machine learning' OR 'machine learning'/exp | 569,215 |
| #3 | surviv* | 2,018,718 |
| #4 | #1 AND #2 AND #3 | 2,678 |
