# Supplementary material for: Predicting breast cancer 5-year survival using machine learning: A systematic review
Source: PLoS One. 2021 Apr 16;16(4):e0250370. doi: 10.1371/journal.pone.0250370 (PMC8051758; doi:10.1371/journal.pone.0250370)
Supplement: S3 Table — (DOCX) [file pone.0250370.s003.docx]

**S3 Table. Primary information of the 31 studies.**

| **Basic information of literature** | | | | | **Basic information of data** | | | |
| --- | --- | --- | --- | --- | --- | --- | --- | --- |
| **First author, year** | **Country under research** | **Published type** | **Disease characters** | **Predict outcome** | **Source of data** | **Type of data** | **Number of centers** | **Number of sample** |
| Delen,2005 | USA | Journal article | Bresat cancer | 5-year survival | SEER (1973-2000) | Public | Multiple center | 202932 |
| Bellaachia,2006 | USA | Information article | Bresat cancer | 5-year survival | SEER (1973-2002) | Public | Multiple center | 151886 |
| Endo,2008 | Japan | Journal article | Bresat cancer | 5-year survival | SEER (1972-1997) | Public | Multiple center | 37256 |
| Khan,2008 | Korea | Journal article | Bresat cancer | 5-year survival | SEER (1973-2003) | Public | Multiple center | 162500 |
| Thongkam,2008 | Australia | Conference paper | Bresat cancer | 5-year survival | Srinagarind Hospital records in Thailand (1990-2001) | Private | Single center | 732 |
| Choi,2009 | Korea | Journal article | Bresat cancer | 5-year survival | SEER (1973-2003) | Public | Multiple center | 294275 |
| Liu,2009 | China | Conference paper | Bresat cancer | 5-year survival | SEER (1973-2004) | Public | Multiple center | 182517 |
| Wang,2013 | China | Journal article | Bresat cancer | 5-year survival | SEER (1973-2007) | Public | Multiple center | 215221 |
| Kim,2013 | Korea | Journal article | Bresat cancer | 5-year survival | SEER (1973-2003) | Public | Multiple center | 162500 |
| Park,2013 | Korea | Journal article | Bresat cancer | 5-year survival | SEER (1973-2003) | Public | Multiple center | 162500 |
| Shin,2014 | Korea | Journal article | Bresat cancer | 5-year survival | SEER (1973-2003) | Public | Multiple center | 162500 |
| Wang,2015 | China | Journal article | Bresat cancer | 5-year survival | Northern Taiwan hospital database (2000-2003) | Private | Single center | 604 |
| Wang,2014 | China | Journal article | Bresat cancer | 5-year survival | SEER (1973-2007) | Public | Multiple center | 215221 |
| Chao,2014 | China | Journal article | Bresat cancer | 5-year survival | Breast cancer incidence database in Taiwan hospital (2002-2010) | Private | Single center | 1340 |
| García-Laencina,2015 | Spain | Journal article | Bresat cancer | 5-year survival | Institute Portuguese of Oncology of Porto | Private | Single center | 399 |
| Lotfnezhad Afshar,2015 | Iran | Journal article | Bresat cancer | 5-year survival | SEER (1999-2004) | Public | Multiple center | 22763 |
| Khalkhali,2016 | Iran | Journal article | Bresat cancer | 5-year survival | Omid Treatment and Research Center database (2007-2010) | Private | Single center | 569 |
| Shawky,2017 | Egypt | Journal article | Bresat cancer | 5-year survival | SEER (2010) | Public | Multiple center | 4490 |
| Sun,2018 | China | Journal article | Bresat cancer | 5-year survival | Molecular Taxonomy of Breast Cancer International Consortium | Public | Multiple center | 1980 |
| Sun,2018 | China | Journal article | Bresat cancer | 5-year survival | the Cancer Genome Atlas | Public | Multiple center | 578 |
| Zhao,2018 | USA | Journal article | Bresat cancer | 5-year survival | Molecular Taxonomy of Breast Cancer International Consortium | Public | Multiple center | 1874 |
| Fu,2018 | China | Journal article | Bresat cancer | 5-year invasive disease free survival | the clinical research center for breast in West China Hospital of Sichuan University (1989-2007) | Private | Single center | 5246 |
| Lu,2019 | USA | Journal article | Bresat cancer | 5-year survival | SEER (1973-2014) | Public | Multiple center | 82707 |
| Abdikenov,2019 | Australia | Journal article | Bresat cancer | 5-year survival | SEER (2004-2014) | Public | Multiple center | 659802 |
| Kalafi,2019 | Malaysia | Journal article | Bresat cancer | 5-year survival | The University Malaya Medical Centre Breast Cancer Registry database (1993-2017) | Private | Single center | 4902 |
| Shouket,2019 | Pakistan | Conference paper | Bresat cancer | 5-year survival;  5-year disease free survival | Institute of Nuclear Medicine & Oncology Lahore Hospital of Pakistan database (2013-2018) | Private | Single center | 200 |
| Ganggayah,2019 | Malaysia | Journal article | Bresat cancer | 5-year survival | The University Malaya Medical Centre Breast Cancer Registry database (1993-2017) | Private | Single center | 8066 |
| Simsek,2020 | USA | Journal article | Bresat cancer | 1-year survival;  5-year survival;  10-year survival | SEER (1973-2013) | Public | Multiple center | 53732 |
| Salehi,2020 | Iran | Journal article | Bresat cancer | 5-year survival | SEER (2004-2013) | Public | Multiple center | 141254 |
| Tang,2020 | China | Journal article | Bresat cancer | 5-year survival | Haberman’s Survival Data Set (1958-1970) | Public | Multiple center | 306 |
| Hussain,2020 | Irap | Journal article | Bresat cancer | 5-year survival | SEER (1973-2001) | Public | Multiple center | 90308 |

Abbreviation: USA=United State of American; SEER=the Surveillance epidemiology and End Result
