# Supplementary material for: Predicting breast cancer 5-year survival using machine learning: A systematic review
Source: PLoS One. 2021 Apr 16;16(4):e0250370. doi: 10.1371/journal.pone.0250370 (PMC8051758; doi:10.1371/journal.pone.0250370)
Supplement: S4 Table — (DOCX) [file pone.0250370.s004.docx]

**S4 Table. Data preparation and modeling process information of the 31 studies.**

| **First author, year** | **Missing data described** | **Missingness processing described** | **Pre-processing algorithms** | **Pre-processing described** | **Feature selection algorithms** | **Feature selection described** | **Class imbalance (Alive + Dead)** | **Number of candidate predictors used** | **Machine Learning Algorithms** | **Model** **presentation** | **Software or environment used** |
| --- | --- | --- | --- | --- | --- | --- | --- | --- | --- | --- | --- |
| Delen,2005 | Yes  16% of the records in the Site  Specific Surgery variable contained missing data. | Yes  Missing data were removed from the dataset. Further analysis showed that missing data was no considerable change in the distribution of the other variables. | No | Yes | No | No | 109659+93273 | 16 | ANN; C5 DT; LR | Formula and graph | Access, SPSS, Statistica data miner, Clementine data mining toolkit |
| Bellaachia,2006 | No  No missing data | No | No | Yes | No | No | 116738+35148 | 16 | Naive Bayes; MLP; C4.5 DT | Formula | WEKA |
| Endo,2008 | No | No | No | Yes | No | No | 30734+6882 | 10 | LR; ANN; Naive Bayes; Bayesian network; DT + Naive Bayes; DT ID3; DT J48 | Graph | WEKA, R |
| Khan,2008 | Yes | Yes  Records containing missing data were removed. | No | Yes | No | No | Unknown | 16 | Fuzzy decision trees | Formula and graph | WEKA, Java |
| Thongkam,2008 | Yes | Yes | Yes  C-Support Vector Classification Filter, AdaBoost, Boosting, AdaBoost SVM, Boosting SVM (Each algorithm removes 5%, 10%, 15%, and 20% outliers respectively) | Yes | No | No | 342+394 | 11 | C4.5 DTs; Conjunctive rule; Naive Bayes; NN-classifier; random committee; RF; Radial basis function network | No presentation | WEKA, LIBSVM |
| Choi,2009 | Yes | Yes  Those variables that had more than 70.0% missing values were removed. | No | Yes | No | No | Unknown | 14 | ANN; Bayesian network; Hybrid Bayesian network | Formula and graph | NETICA |
| Liu,2009 | Yes | Yes  The missing values were removed. | Yes  Under-sampling, bagging algorithm | Yes  The abnormal values and incorrect values were removed. | Yes | Yes  Using implemented with LR backward selection. | 157916+24601 | 16 | C5 DT | No presentation | Unknown |
| Wang,2013 | Yes | Yes | Yes  SMOTE,  CSC,  under-sampling, bagging, boosting, AdaboostM1 | Yes | The correlation-based feature selection method | Yes | 195172+20049 | 9 | DT; LR | Formula | Unknown |
| Kim,2013 | No | No | No | Yes | No | No | 128469+34031 | 16 | SVM; ANN; Semi-supervised learning; Semi-supervised learning-Co training | Formula and graph | Unknown |
| Park,2013 | No | No | No | Yes | No | No | 128469+34031 | 16 | SVM; ANN; Semi-supervised learning | Formula and graph | MATLEB |
| Shin,2014 | No | No | No | Yes | No | No | 128469+34031 | 16 | DT; ANN; SVM; Semi-supervised learning; Semi-supervised learning-Co training | Formula and graph | Unknown |
| Wang,2015 | Yes | Yes | No | Yes | No | Yes.  The feature selection  was based on literature review and clinical availability | 464+140 | 5 | ANN | No presentation | STATISTICA |
| Wang,2014 | No | No | Yes  SMOTE, PSO | Yes | No | No | 195172+20049 | 20 | LR; C5 DT; 1-nearest neighbor | Formula and graph | Visual studio c++ |
| Chao,2014 | No | No | No | Yes | No | No | 1272+68 | 7 | SVM; LR; C5 DT | Formula | SPSS, Clementine |
| García-Laencina,2015 | Yes | Yes  M imp, EM imp, KNN imp | Yes | Yes | No | No | 282+117 | 16 | KNN; Classification Trees; LR; SVM | Formula | Unknown |
| Lotfnezhad Afshar,2015 | Yes | Yes  MI | Yes  Under-sampling or over-sampling | Yes | No | No | Aliveness values were approximately nine times greater  than death values | 18 | SVM; Bayesian network; CHi-squared Automatic Interaction Detection | No presentation | Access, Excel, SPSS |
| Khalkhali,2016 | Yes | Yes  MI | Yes | Yes | No | No | Unknown | 15 | Classification and regression tree | Graph | SPSS |
| Shawky,2017 | Yes | Yes | No | Yes | No | No | 2245+2245 | 14 | ANN; KNN; SVM; LR | Formula | Unknown |
| Sun,2018 | Yes | Yes  weighted nearest neighbors algorithm | Yes | Yes | Yes  minimal-redundancy-maximal-relevance | Yes | 1489+491 | 3 types | SVM; RF; LR; DNN | Formula and graph | TensorFlow, Python Scikit-learn, LIBSVM |
| Sun,2018 | No | No | No | Yes | Yes  information gain ratio measure | Yes | 133+445 | 5 types | Multiple kernel learning; | Formula and graph | R, Fselector |
| Zhao,2018 | Yes | Yes | Yes  K-means,  KNN | Yes | No | No | 1409+465 | 27 | Regular Cox models; Parametric censored regression models; Random survival forests; Boosting concordance index; | Formula and graph | R |
| Fu,2018 | Yes | Yes  Where outliers are evident, necessary information is missing, or data inconsistencies are occurred in the raw data, corrections are made by rechecking and revisiting patients by telephone. | No | Yes | Yes  EFS, SFS | Yes | 1181+4065 | 23 | Supervised principal components regression | Formula and graph | Unknown |
| Lu,2019 | Yes | Yes  The features with more than half of missing records, and the data with a large portion of missing data for selected features was removed. | No | Yes | No | No | 76716+5991 | 14 | Gradient Boosting; RF; SVM; ANN | Formula | Unknown |
| Abdikenov,2019 | No | No | No | Yes | No | No | 376087+283715 | 19 | Gradient Boosting; DT framework; SVM; RF; Adaboost; Cox Regression | Formula and graph | TensorFlow |
| Kalafi,2019 | Yes | Yes  All patients with missing values were  removed. | No | Yes | No | No | 2451+2451 | 23 | Genetic algorithm-based online gradient; Online Sequential Extreme Learning Machine; Online Adaptive Boosting with the Adaptive Linear Regressor; Online Gradient Boosting with the Adaptive Linear Regressor; Online linear regressor; AdaBoost; SVM; MLP | Formula and graph | Python Scikit-learn |
| Shouket,2019 | No | No | Yes  Over sampled | Yes | No | No | 5-year survival:190+10;  5-year DFS:164+36 | 10 | DNN; LR; SVM; RF; Gradient Boosting | Graph | Unknown |
| Ganggayah,2019 | Yes | Yes | No | Yes | Yes  Threshold-based pre-selection method and clustered. | Yes  Adopting the threshold-based pre-selection method. All data and clustered datasets were used for feature selection. | 5614+2452 | 23 | SVM; RF; DT; MLP | Graph | R, Python |
| Simsek,2020 | Yes | Yes | Yes  RUS, SMOTE | Yes | Yes  GA, LASSO | Yes | 1-year:52886+886; 5-year:46724+7028; 10-year:42965+10787. | 17 | Naive Bayes; DT J48; SVM; RF; AdaBoost; JRip | Formula and graph | R |
| Salehi,2020 | Yes | Yes  those features having more than 50% of missing values and those features with an equal value in more than 90% of records (such as gender, because more than 98% of data are related to females) were removed. In addition, those records which had missing values in the main features, such as stage, were removed. | No | Yes | No | No | 118324+22930 | 35 | DT (rpart); RF; NN; XGboost; LR; SVM | Formula and graph | Unknown |
| Tang,2020 | No | No | Yes  min–max normalization rule; GABC algorithm | Yes | No | No | Unknown | 3 | ANN; LR | Formula and graph | Unknown |
| Hussain,2020 | Yes | Yes | No | Yes | No | No | Unknown | 17 | MLP; MLP-experts; MLP stacked generalization | Graph | SAS |

Abbreviation: ANN=artificial neural network; DT=decision trees; LR=logistic regression; MLP=multi-layer perceptron; KNN=K-nearest neighbors; SVM=support vector machines; RF=random forest; DNN=deep neural network; SMOTE=Synthetic minority over-sampling technique; CSC=cost-sensitive classifier technique; PSO=particle swarm optimization; M imp=mean/mode imputation; EM imp=Expectation-Maximization imputation; KNN imp=KNN imputation; MI=multiple imputation; EFS=Ensemble Feature Selection; SFS=Stratified Feature Selection; GA=Genetic algorithm, LASSO=The Least Absolute Shrinkage and Selectionator operator; RUS=random under-sampling; GABC=gbest-guided artificial bee colony
