# Supplementary material for: Predicting breast cancer 5-year survival using machine learning: A systematic review
Source: PLoS One. 2021 Apr 16;16(4):e0250370. doi: 10.1371/journal.pone.0250370 (PMC8051758; doi:10.1371/journal.pone.0250370)
Supplement: S5 Table — (DOCX) [file pone.0250370.s005.docx]

**S5 Table. Model construction and performance evaluation information of the 31 studies.**

| **First author, year** | **Internal validation** | **External validation** | **Model evaluation metrics** | **Calibration metrics** | **Hyperparameter tuning** | **Discrimination and classification metrics** |
| --- | --- | --- | --- | --- | --- | --- |
| Delen,2005 | 10-fold cross validation | No | Accuracy; Sensitivity; Specificity | No | No | Mean (SD)  ANN: accuracy=0.9121(0.0111); sensitivity=0.9437(0.0131); specificity=0.8748(0.0135).  LR: accuracy=0.8920(0.0020); sensitivity=0.9017(0.0014); specificity=0.8786(0.0038).  DT C5: accuracy=0.9362(0.0016); sensitivity=0.9602(0.0019); specificity=0.9066(0.0028). |
| Bellaachia,2006 | 10-fold cross validation | No | Accuracy;  Sensitivity(recall);  Precision | No | No | NB: Accuracy(%)=84.5; Class=0: Precision=0.70; Recall=0.57 ; Class=1: Precision=0.88; Recall=0.93. ANN: Accuracy(%)=86.5; Class=0; Precision=0.83; Recall=0.52; Class=1: Precision=0.87; Recall=0.97. C4.5 DT: Accuracy(%)=86.7; Class=0; Precision=0.80; Recall=0.56; Class=1: Precision=0.88; Recall=0.96. |
| Endo,2008 | 10-fold cross validation | No | Accuracy; Sensitivity; Specificity | No | No | LR: Sensitivity=97.0%; Specificity=36.3%; Accuracy(95%CI)=85.8%(85.6%-86.0%). DT J48: Sensitivity=97.1%; Specificity=34.7%; Accuracy(95%CI)=85.6%(85.4%- 85.8%). DT+NB: Sensitivity=92.7%; Specificity=46.4%; Accuracy(95%CI)=84.2%(84.0%- 84.4%). ANN: Sensitivity=92.2%; Specificity=50.9%; Accuracy(95%CI)=84.5%(83.1%-85.9%). NB: Sensitivity=92.3%; Specificity=47.1%; Accuracy(95%CI)=83.9%(83.7%-84.1%); BN: Sensitivity=92.3%; Specificity=47.1 %; Accuracy(95%CI)=83.9%(83.7%-84.1%). DT ID3: Sensitivity=91.6%; Specificity=40.9%; Accuracy(95%CI)=82.3%(82.1%-82.5%). |
| Khan,2008 | Out of 162500 records, taking into account the overlapping factor, 30000 records as training and 10600 as test data were obtained using uniform random selection | No | Accuracy; Sensitivity; Specificity | No | No | DTs: Yes: Sensitivity=0.95; Specificity=0.34; Accuracy=82%; No: Sensitivity=0.35; Specificity=0.96; Accuracy=82%.  FDTs: Yes: Sensitivity=0.98; Specificity=0.39; Accuracy=85%; No: Sensitivity=0.39; Specificity=0.99; Accuracy=85%. |
| Thongkam,2008 | 10-fold cross validation | No | Accuracy; AUC | No | No | Accuracy: Optimal outlier removal model: DT C4.5=85.91%; Conjunctive Rule=82.00%; NB=84.38%; NN-Classifiers=82.00%; Random Committee=83.53%; RF=83.53%; RBF Network=84.04%. AUC: Optimal outlier removal model: DT C4.5=85.60%; Conjunctive Rule=80.50%; NB=89.10%; NN-Classifiers=81.60%; Random Committee=89.20%; RF=90.80%; RBF Network=89.00%. |
| Choi,2009 | 10-fold cross validation, train sample: test sample=5:5 | No | Accuracy; Sensitivity; Specificity; AUC | No | No | ANN: Sensitivity(SD)=93.7%(1.0%); Specificity(SD)=85.4%(0.6%); Accuracy(SD) =88.8%(0.4%); AUC(SD)=0.930(0.012). BN: Sensitivity(SD)=88.5%(0.1%); Specificity(SD)=58.3%(0.1%); Accuracy(SD)= 70.9% (0.1%); AUC(SD)=0.813(0.002). Hybrid BN: Sensitivity(SD)=93.3%(4.7%); Specificity(SD)=83.1%(5.0%); Accuracy(SD) =87.2%(1.7%); AUC(SD)=0.935(0.009). |
| Liu,2009 | 10-fold cross validation, train sample: test sample=1:9 | No | Accuracy; Sensitivity; Specificity; AUC | No | No | C5(DT): AUC=0.6070; specificity=0.2325; sensitivity=0.9814; accuracy=0.8805.  C5(DT) with under-sampling ratio of 15%: AUC=0.7484; specificity=0.7570; the sensitivity=0.7399; accuracy=0.7422.  C5(DT) +bagging algorithm: AUC=0.7678; specificity=0.7859; sensitivity=0.7496; accuracy=0.7659. |
| Wang,2013 | 10-fold cross validation, train sample: test sample=9:1 | No | Accuracy; Sensitivity; Specificity; AUC;  G-mean | No | No | Optimal performance model: C_LR_9: Accuracy=0.752; Sensitivity=0.752; Specificity=0.752; G-mean=0.752; AUC=0.829. |
| Kim,2013 | 5-fold cross validation,  the equipoise  dataset of 50 000 data points was eventually divided into  10 groups, train sample: test sample=4:1 | No | Accuracy; AUC | No | No | ANN: Accuracy=0.65; AUC=0.70.  SVM: Accuracy=0.51; AUC=0.80.  SSL: Accuracy=0.70; AUC=0.78.  SSL Co-training: Accuracy=0.76; AUC=0.81. |
| Park,2013 | 5-fold cross validation,  The equipoise  dataset of 50 000 data points was eventually divided into  10 groups, train sample: test sample=4:1 | No | Accuracy; Sensitivity; Specificity; AUC | No | Yes | Best performance model (Mean (±SD)):  ANN: Accuracy=0.65(±0.02); Sensitivity=0.73(±0.11); Specificity=0.58(±0.10); AUC=0.70(±0.03).  SVM: Accuracy=0.51(±0.01); Sensitivity=0.65(±0.13); Specificity=0.52(±0.02); AUC=0.80(±0.01).  SSL: Accuracy=0.71(±0.02); Sensitivity=0.76(±0.03); Specificity=0.65(±0.03); AUC=0.78(±0.01). |
| Shin,2014 | 5-fold cross validation,  The equipoise  dataset of 50 000 data points was eventually divided into  10 groups, train sample: test sample=4:1 | No | AUC | No | Yes | DT: AUC=0.73.  ANN: AUC=0.70.  SVM: AUC=0.80.  SSL: AUC=0.78.  SSL-Co training: AUC=0.81. |
| Wang,2015 | train sample: test sample=3:1 | No | Accuracy; Sensitivity; Specificity; AUC | No | No | ANN: Accuracy=85.1%; Sensitivity=94.83%; Specificity=52.86%; AUC=0.7935. |
| Wang,2014 | 10-fold cross validation  train sample: test sample=9:1 | No | Accuracy; Sensitivity; Specificity; G-mean | No | Yes | LR: G-mean=0.417; Sensitivity=0.945; Specificity=0.184; Accuracy=91.495. PSO + LR : G-mean=0.421; Sensitivity=0.962; Specificity=0.184 ; Accuracy=91.293. SMOTE + PSO + LR : G-mean=0.764; Sensitivity=0.783; Specificity=0.746; Accuracy=74.267. C5 DT : G-mean=0.390; Sensitivity=0.926; Specificity=0.165; Accuracy=90.271. PSO + C5 DT : G-mean=0.483; Sensitivity=0.946; Specificity=0.248 ; Accuracy=89.142. SMOTE + PSO + C5 DT : G-mean=0.959; Sensitivity=0.974; Specificity=0.945; Accuracy=94.255. 1-nn : G-mean=0.484; Sensitivity=0.949; Specificity=0.248; Accuracy=86.459. PSO + 1-nn :G-mean=0.517; Sensitivity=0.931; Specificity=0.290; Accuracy=86.662. SMOTE + PSO + 1-nn :G-mean=0.802; Sensitivity=0.756; Specificity=0.852 ; Accuracy=87.353. |
| Chao,2014 | stratified 10-fold cross validation | No | Accuracy | No | Yes | Mean(SD)  SVM: Accuracy=95.22 (2.52) %.  LR: Accuracy=95.1 (2.43) %.  C5 DT: Accuracy=93.95% (2.54) %. |
| García-Laencina,2015 | 10-fold cross validation | No | Accuracy; Sensitivity; Specificity; AUC | No | Yes  2-D grid search | KNN + M imp: AUC =0.7321(4.56e-2); Accuracy (%)=78.08(3.63); Sensitivity (%)=85.08(3.66); Specificity (%)=61.33(5.74).  KNN + EM imp: AUC =0.7810(2.82e-2); Accuracy (%)=81.46(2.34); Sensitivity (%)=85.77(2.48); Specificity (%)=70.43(3.98).  KNN + KNN imp: AUC =0.7845(2.88e-2); Accuracy (%)=81.73(2.32); Sensitivity (%)=86.44(2.66); Specificity (%)=70.46(3.36).  CT + M imp: AUC =0.6563(3.22e-2); Accuracy (%)=68.83(2.70); Sensitivity (%)=83.86(3.14); Specificity (%)=47.39(3.52).  CT + EM imp: AUC =0.6772(2.14e-2); Accuracy (%)=71.06(1.77); Sensitivity (%)=85.12(2.33); Specificity (%)=50.31(2.17).  CT + KNN imp: AUC =0.7012(3.32e-2); Accuracy (%)=74.27(2.77); Sensitivity (%)=85.79(3.24); Specificity (%)=54.42(3.63).  LR + M imp: AUC =0.7164(3.13e-2); Accuracy (%)=75.25(2.84); Sensitivity (%)=87.20(3.14); Specificity (%)=56.07(3.64).  LR + EM imp: AUC =0.7373(2.89e-2); Accuracy (%)=77.21(2.66); Sensitivity (%)=88.17(3.01); Specificity (%)=59.30(3.80).  LR + KNN imp: AUC =0.7514(2.99e-2); Accuracy (%) =79.08(2.65); Sensitivity (%) =88.04(3.03); Specificity (%)=62.23(3.50).  SVM + M imp: AUC =0.7168(2.80e-2); Accuracy (%) =75.39(2.53); Sensitivity (%) =86.84(3.00); Specificity (%)=56.52(3.34).  SVM + EM imp: AUC =0.7348(3.17e-2); Accuracy (%) =77.30(2.73); Sensitivity (%) =88.38(3.22); Specificity (%)=58.59(3.32).  SVM + KNN imp: AUC =0.7540(3.34e-2); Accuracy (%) =79.6(2.88)1; Sensitivity (%) =87.24(3.08); Specificity (%)=63.55(3.91). |
| Lotfnezhad Afshar,2015 | train sample: test sample=7:3 | No | Accuracy; Sensitivity; Specificity; Adjusted propensity | No | No | SVM: Sensitivity=97.7%; Specificity=95.6%; Accuracy=96.7 %; Adjusted Propensity scores=0.977. BN: Sensitivity=81.8%; Specificity=86.1%; Accuracy=83.9%; Adjusted Propensity scores=0.880. CHAID: Sensitivity=82.2%; Specificity=82.7%; Accuracy=82.4%; Adjusted Propensity scores=0.829. |
| Khalkhali,2016 | 10-fold cross validation | No | Accuracy; Sensitivity; Specificity | No | No | CART: Sensitivity=93.5%; Specificity=53%; Accuracy=80.3%. |
| Shawky,2017 | k-fold cross validation | No | Accuracy; Sensitivity; Specificity; AUC | No | No | Mean(SD)  ANN:RCV:AUC=0.8402(2.21e-2); Accuracy (%)=88.928(1.68); Sensitivity (%)=87.35(2.03); Specificity (%)=85.01(1.27);RV:AUC=0.8100(2.32e-2);Accuracy (%)=82.46(1.52); Sensitivity (%)=83.77(2.48); Specificity (%)=81.73(1.59). KNN:RCV:AUC=0.8372(2.05e-2); Accuracy (%)=88.11(2.37); Sensitivity (%)=85.86(1.16); Specificity (%)=84.31(1.25); RV:AUC=0.8001(2.31e-2); Accuracy (%)=81.06(1.77); Sensitivity (%)=80.15(0.97); Specificity (%)=78.53(1.67). SVM:RCV:AUC=0.8233(1.25e-2); Accuracy (%)=85.35(2.52); Sensitivity (%)=82.20(2.15); Specificity (%)=81.07(2.37); RV:AUC=0.7910(1.81e-2); Accuracy (%)=80.33(2.12); Sensitivity (%)=79.17(3.01); Specificity (%)=77.30(2.40). LR:RCV:AUC=0.7351(2.20e-2); Accuracy (%)=77.35(1.20); Sensitivity (%)=76.52(2.10); Specificity (%)=74.52(1.82); RV:AUC=0.6279(1.17e-2); Accuracy (%)=67.55(1.78); Sensitivity (%)=66.58(2.22); Specificity (%)=63.55(1.27). |
| Sun,2018 | 10-fold cross validation, train sample: test sample=8:2 | yes  Downloading 1,054 valid breast cancer patients from TCGA project | Accuracy; Sensitivity; Precision; Specificity; AUC;  Mcc | No | Yes | DNN: Accuracy=0.794; Precision=0.875; Sensitivity=0.200; Specificity=0.990; Mcc=0.356. SVM: Accuracy=0.775; Precision=0.811; Sensitivity=0.122; Specificity=0.990; Mcc=0.257. RF: Accuracy=0.770; Precision=0.787; Sensitivity=0.098; Specificity=0.990; Mcc=0.223. LR: Accuracy=0.754; Precision=0.563; Sensitivity=0.037; Specificity=0.990; Mcc=0.093.  DNN: Accuracy=0.826; Precision=0.749; Sensitivity=0.450; Specificity=0.950; Mcc=0.486. SVM: Accuracy=0.805; Precision=0.708; Sensitivity=0.365; Specificity=0.950; Mcc=0.407. RF: Accuracy=0.791; Precision=0.766; Sensitivity=0.226; Specificity=0.950; Mcc=0.337. LR: Accuracy=0.760; Precision=0.549; Sensitivity=0.183; Specificity=0.950; Mcc=0.209. |
| Sun,2018 | k-fold cross validation | No | Accuracy;  Sensitivity;  Specificity;  Precision;  AUC;  Mcc;  C-index | No | No | geneExpr-kernel: AUC=0.705.  CNA-kernel: AUC=0.534.  methyl-kernel: AUC=0.731.  protein-kernel: AUC=0.720.  GMKL: AUC=0.794.  PMKL: AUC=0.681.  GPMKL: AUC=0.821.  geneExpr+CNA+methyl+protein+image: AUC=0.821.  CNA+methyl+ protein+image: AUC=0.788.  geneExpr+methyl+protein+image: AUC=0.812.  geneExpr+CNA+protein+image: AUC=0.799.  geneExpr+CNA+methyl+image: AUC=0.780.  geneExpr+image: AUC=0.746.  CNA+image: AUC=0.690.  methyl+image: AUC=0.760.  protein+image: AUC=0.756.  image: AUC=0.681.  At the high level of specificity (specificity = 95.0%), precision, accuracy, sensitivity and Mcc values of GPMKL are increased by 3.8%, 0.9%, 4.3% and 4.7% compared with GMKL and have an improvement of 16.4%, 3.2%, 13.9% and 16.7% compared with PMKL, respectively. In addition, when specificity decreases into 90.0%, the sensitivity value of the proposed method is 0.285, while the corresponding sensitivity values of geneExpr-kernel, CNA-kernel, methyl- kernel, and protein-kernel are 0.118, 0.049, 0.133 and 0.144, respectively. The precision values of GPMKL, GMKL, PMKL, geneExpr- kernel, CNA-kernel, methyl-kernel, and protein-kernel are 0.634, 0.596, 0.470, 0.418, 0.217, 0.447 and 0.467, respectively.  LASSO-Cox: Genomic data: AUC=0.697 ±0.069; Pathological image: AUC=0.655 ±0.059; Genomic data + pathological image: AUC=0.698 ±0.060; C-index=0.612.  En-Cox: Genomic data: AUC=0.667 ±0.080; Pathological image: AUC=0.649 ±0.056; Genomic data + pathological image: AUC=0.677 ±0.067; C-index=0.602.  PCRM: Genomic data: AUC=0.620 ±0.067; Pathological image: AUC=0.608 ±0.055; Genomic data + pathological image: AUC=0.546 ±0.043; C-index=0.531.  RSF: Genomic data: AUC=0.722 ±0.049; Pathological image: AUC=0.620 ±0.067; Genomic data + pathological image: AUC=0.718 ±0.055; C-index=0.617.  BoostCI: Genomic data: AUC=0.716 ±0.037; Pathological image: AUC=0.622 ±0.058; Genomic data + pathological image: AUC=0.717 ±0.037; C-index=0.598.  superPC: Genomic data: AUC=0.659 ±0.069; Pathological image: AUC=0.595 ±0.056; Genomic data + pathological image: AUC=0.698 ±0.048; C-index=0.605.  GPMKL: Genomic data: AUC=0.802 ±0.032; Pathological image: AUC=0.690 ±0.046; Genomic data + pathological image: AUC=0.828 ±0.034; C-index=0.643. |
| Zhao,2018 | 10-fold cross validation, train sample: test sample=8:2 | No | Accuracy; AUC; | No | No | GB:AUC (95% CI)=0.669 (0.608, 0.730); Accuracy (95% CI)=0.697 (0.648, 0.743). RF:AUC (95% CI)=0.677 (0.617, 0.736); Accuracy (95% CI)=0.729 (0.681, 0.773). SVM:AUC (95% CI)=0.658 (0.596, 0.720); Accuracy (95% CI)=0.729 (0.681, 0.773). ANN:AUC (95% CI)=0.673 (0.611, 0.735); Accuracy (95% CI)=0.721 (0.672, 0.765). |
| Fu,2018 | 10-fold cross validation, train sample: test sample=8:2 | No | cut-off; Youden index; Sensitivity; Specificity; F-score;  AUC | No | Yes  The TPE algorithm | MP4Ei: cut-off=0.235;Youden index=0.535;Sensitivity=0.742;Specificity=0.794;F-score=0.605;AUC=0.8451. RF: cut-off=0.305;Youden index=0.495;Sensitivity=0.653;Specificity=0.843;F-score=0.595;AUC=0.8192. Adaboost: cut-off=0.498;Youden index=0.499;Sensitivity=0.814;Specificity=0.686;F-score=0.561;AUC=0.8196. SVM: cut-off=0.231;Youden index=0.469;Sensitivity=0.665;Specificity=0.803;F-score=0.568;AUC=0.7912. Cox: cut-off=0.189;Youden index=0.457;Sensitivity=0.784;Specificity=0.673;F-score=0.539;AUC=0.7857. |
| Lu,2019 | 3-fold cross validation | No | Accuracy; Sensitivity; Specificity; AUC; Retraining Time | No | Yes | Mean(SD) GAOGB: Accuracy (%)=75.0(0.36); AUC (%)=75.07(0.34); Specificity (%)=68.77(0.50) ;Sensitivity (%)=81.36(0.23); Retraining Time (Sec.)=3.80(0.01). OSELM: Accuracy (%)=71.85(0.64) ; AUC (%)=71.84(0.63) ; Specificity (%)=69.77(2.72) ;Sensitivity (%)=73.92(2.76) ; Retraining Time (Sec.)=0.79(0.05). OLRAB: Accuracy (%)=70.31(1.35) ; AUC (%)=70.31(1.34) ; Specificity (%)=62.31(1.40) ;Sensitivity (%)=78.30(1.60) ; Retraining Time (Sec.)=0.19(0.01). OLRGB: Accuracy (%)=73.80(0.21) ; AUC (%)=73.80(0.21) ; Specificity (%)=67.47(0.17) ;Sensitivity (%)=80.54(0.30) ; Retraining Time (Sec.)=0.49(0.86). OLR: Accuracy (%)=53.72(2.75) ; AUC (%)=53.70(2.73) ; Specificity (%)=52.19(2.76) ;Sensitivity (%)=55.21(2.97) ; Retraining Time (Sec.)=0.02(0.00). |
| Abdikenov,2019 | 10-fold cross validation | No | Accuracy; Sensitivity; Specificity; AUC;  F1 score | No | Yes  NSGAIII | DNN: Accuracy=97.09;Sensitivity=0.9663;Specificity=0.9689;F1 Score=0.9646;AUC=97.15. LR: Accuracy=96.99;Sensitivity=0.9719;Specificity=0.9685;F1 Score=0.9651;AUC=97.02. SVM: Accuracy=97.02;Sensitivity=0.9677;Specificity=0.9720;F1 Score= 0.9652;AUC=96.98. RF: Accuracy=97.07;Sensitivity=0.9787;Specificity=0.9647;F1 Score=0.9662;AUC=97.17. GB: Accuracy=97.08;Sensitivity=0.9771;Specificity=0.9661;F1 Score=0.9662;AUC=97.16. |
| Kalafi,2019 | 10-fold cross validation,  train sample: test sample: valid sample=18:1:1 | No | Accuracy; Sensitivity; Precision; Specificity; F1 score;  Mcc;  NPV;  FPR;  FDR;  FNR; | No | No | MLP: Sensitivity=0.960; Specificity=0.830; Precision=0.792; NPV=0.968 ; FPR=0.17; FDR=0.208; FNR=0.040; Accuracy =0.882; F1 Score=0.868; Mcc=0.775.  DT: Sensitivity=1.000; Specificity=0.739; Precision=0.653; NPV=1.000; FPR=0.261; FDR=0.347; FNR=0.000; Accuracy =0.825; F1 Score=0.79; Mcc=0.695.  RF: Sensitivity=0.937; Specificity=0.768; Precision=0.718 ; NPV=0.951; FPR=0.232; FDR=0.282; FNR=0.063; Accuracy =0.833; F1 Score=0.813; Mcc=0.687.  SVM: Sensitivity=0.977; Specificity=0.709; Precision=0.652; NPV=0.983; FPR=0.291; FDR=0.349; FNR=0.023; Accuracy =0.805; F1 Score=0.782; Mcc=0.66. |
| Shouket,2019 | 10-fold cross validation | No | Accuracy; Precision;  NPV;  AUC;  F-score;  Mcc | No | No | 5-year survival: JRip: Accuracy=96.25%; Precision=0.989;NPV=0.860;F- score=0.962;Mcc=0.884;AUC=0.87. RF: Accuracy=95.83%; Precision=0.984;NPV=0.860;F- score=0.958;Mcc=0.871;AUC=0.952. AdaBoost: Accuracy=95.41%; Precision=0.984;NPV=0.840;F- score=0.953;Mcc=0.858;AUC=0.929. SVM: Accuracy=94.58%; Precision=0.974;NPV=0.840;F- score=0.945;Mcc=0.833;AUC=0.907. NB: Accuracy=92.5%; Precision=0.937;NPV=0.88 ;F- score=0.927;Mcc=0.784;AUC=0.958. J48:Accuracy=79.16%; Precision=1;NPV=0;F- score=0.7;Mcc=0;AUC=0.5.  5-year Disease free Survival Rate (DFS): JRip: Accuracy=85.38%; Precision=0.90;F- score=0.853;Mcc=0.75;AUC=0.881. RF: Accuracy=84.37%; Precision=0.87;F- score=0.847;Mcc=0.74;AUC=0.938. SVM: Accuracy=82.46%; Precision=0.82;F- score=0.825;Mcc=0.71;AUC=0.871. J48:Accuracy=82.46%; Precision=0.82;F- score=0.826;Mcc=0.72;AUC=0.859. NB: Accuracy=79.87%; Precision=0.76;F- score=0.802;Mcc=0.69;AUC=0.936. AdaBoost: Accuracy=52.92%; Precision=0.99;F- score=0.369;Mcc=-0.02;AUC=0.628. |
| Ganggayah,2019 | train sample: test sample=7:3 | No | Accuracy; Sensitivity; Specificity; AUC; Precision; Mcc | Yes  Calibration curve | No | DT: Accuracy (%)=79.80;Sensitivity=0.82;Specificity =0.75;AUC=0.72;Precision=0.91;Mcc=0.52. RF: Accuracy (%)=82.70;Sensitivity=0.83;Specificity =0.81;AUC=0.86;Precision=0.93;Mcc=0.59. NN: Accuracy (%)=82.00;Sensitivity=0.83;Specificity =0.79;AUC=0.84;Precision=0.93;Mcc=0.58. Extreme boost: Accuracy (%)=81.70;Sensitivity=0.84;Specificity =0.75;AUC=0.87;Precision=0.89;Mcc=0.57. LR: Accuracy (%)=81.10;Sensitivity=0.82;Specificity =0.78;AUC=0.85;Precision=0.92;Mcc=0.55. SVM: Accuracy (%)=81.80;Sensitivity=0.81;Specificity =0.84;AUC=0.85;Precision=0.95;Mcc=0.57. |
| Simsek,2020 | 10-fold cross validation | No | Accuracy; Sensitivity; Specificity; AUC; | No | No | LR:  1-year:  LASSO(RUS):Accuracy=0.840(0.003);Sensitivity=0.692(0.034);Specificity=0.842(0.003);AUC=0.842(0.024); LASSO(SMOTE):Accuracy=0.838(0.003);Sensitivity=0.693(0.038);Specificity=0.841(0.004);AUC=0.845(0.026); GA(RUS):Accuracy=0.741(0.006);Sensitivity=0.748(0.045);Specificity=0.741(0.007);AUC=0.818(0.020); GA(SMOTE):Accuracy=0.819(0.011);Sensitivity=0.756(0.060);Specificity=0.820(0.012);AUC=0.861(0.024); LASSO+GA(RUS):Accuracy=0.737(0.005);Sensitivity=0.788(0.46);Specificity=0.736(0.005);AUC=0.829(0.018); LASSO+GA(SMOTE):Accuracy=0.807(0.007);Sensitivity=0.789(0.050);Specificity=0.808(0.008);AUC=0.870(0.022); 5-year:  LASSO(RUS):Accuracy=0.726(0.006);Sensitivity=0.751(0.010);Specificity=0.722(0.007);AUC=0.815(0.006); LASSO(SMOTE):Accuracy=0.749(0.005);Sensitivity=0.731(0.006);Specificity=0.752(0.006);AUC=0.820(0.006); GA(RUS):Accuracy=0.746(0.006);Sensitivity=0.740(0.014);Specificity=0.747(0.007);AUC=0.824(0.006); GA(SMOTE):Accuracy=0.757(0.006);Sensitivity=0.737(0.011);Specificity=0.760(0.006);AUC=0.828(0.005); LASSO+GA(RUS):Accuracy=0.747(0.006);Sensitivity=0.752(0.013);Specificity=0.734(0.007);AUC=0.824(0.005); LASSO+GA(SMOTE):Accuracy=0.755(0.006);Sensitivity=0.738(0.012);Specificity=0.757(0.007);AUC=0.829(0.005); 10-year:  LASSO(RUS):Accuracy=0.724(0.007);Sensitivity=0.692(0.011);Specificity=0.732(0.009);AUC=0.785(0.007); LASSO(SMOTE):Accuracy=0.736(0.006);Sensitivity=0.694(0.015);Specificity=0.747(0.007);AUC=0.791(0.007); GA(RUS):Accuracy=0.728(0.007);Sensitivity=0.702(0.012);Specificity=0.735(0.009);AUC=0.789(0.007); GA(SMOTE):Accuracy=0.737(0.007);Sensitivity=0.693(0.014);Specificity=0.749(0.009);AUC=0.794(0.007); LASSO+GA(RUS):Accuracy=0.726(0.007);Sensitivity=0.702(0.013);Specificity=0.732(0.008);AUC=0.790(0.007); LASSO+GA(SMOTE):Accuracy=0.738(0.006);Sensitivity=0.695(0.015);Specificity=0.749(0.007);AUC=0.796(0.007).  ANN:  1-year:  LASSO(RUS):Accuracy=0.828(0.011);Sensitivity=0.694(0.034);Specificity=0.830(0.012);AUC=0.840(0.021); LASSO(SMOTE):Accuracy=0.822(0.013);Sensitivity=0.714(0.040);Specificity=0.824(0.013);AUC=0.845(0.021); GA(RUS):Accuracy=0.736(0.006);Sensitivity=0.777(0.005);Specificity=0.735(0.006);AUC=0.826(0.025); GA(SMOTE):Accuracy=0.819(0.010);Sensitivity=0.777(0.051);Specificity=0.820(0.010);AUC=0.869(0.019); LASSO+GA(RUS):Accuracy=0.722(0.013);Sensitivity=0.801(0.049);Specificity=0.721(0.012);AUC=0.829(0.026); LASSO+GA(SMOTE):Accuracy=0.822(0.009);Sensitivity=0.772(0.046);Specificity=0.823(0.009);AUC=0.871(0.020); 5-year:  LASSO(RUS):Accuracy=0.744(0.006);Sensitivity=0.745(0.010);Specificity=0.744(0.007);AUC=0.823(0.006); LASSO(SMOTE):Accuracy=0.761(0.008);Sensitivity=0.743(0.015);Specificity=0.764(0.010);AUC=0.835(0.006); GA(RUS):Accuracy=0.744(0.007);Sensitivity=0.748(0.012);Specificity=0.743(0.008);AUC=0.824(0.006); GA(SMOTE):Accuracy=0.755(0.005);Sensitivity=0.758(0.014);Specificity=0.754(0.007);AUC=0.835(0.005); LASSO+GA(RUS):Accuracy=0.737(0.007);Sensitivity=0.758(0.017);Specificity=0.734(0.009);AUC=0.824(0.006); LASSO+GA(SMOTE):Accuracy=0.756(0.003);Sensitivity=0.755(0.013);Specificity=0.759(0.005);AUC=0.836(0.006); 10-year:  LASSO(RUS):Accuracy=0.726(0.007);Sensitivity=0.706(0.016);Specificity=0.731(0.010);AUC=0.792(0.008); LASSO(SMOTE):Accuracy=0.745(0.006);Sensitivity=0.695(0.015);Specificity=0.758(0.008);AUC=0.803(0.007); GA(RUS):Accuracy=0.721(0.007);Sensitivity=0.710(0.013);Specificity=0.723(0.010);AUC=0.791(0.007) GA(SMOTE):Accuracy=0.741(0.007);Sensitivity=0.706(0.011);Specificity=0.751(0.008);AUC=0.802(0.007); LASSO+GA(RUS):Accuracy=0.724(0.006);Sensitivity=0.705(0.012);Specificity=0.729(0.008);AUC=0.790(0.008); LASSO+GA(SMOTE):Accuracy=0.742(0.011);Sensitivity=0.697(0.016);Specificity=0.754(0.013);AUC=0.801(0.007). |
| Salehi,2020 | 10-fold cross validation | No | Accuracy; Sensitivity; Specificity; | No | No | Predictor’s results: MLP: Sensitivity(%)=94.88(3.12);Specificity(%)=47.74(18.1);Accuracy(%)=81.73(3.09). MLP stacked generalization: Sensitivity(%)=94.34(3.18);Specificity(%)=53.45(17.01);Accuracy(%)=84.59(3.83). Mixture of MLP experts: Sensitivity(%)=95.52(2.9);Specificity(%)=44.59(16.64);Accuracy(%)=79.39(4.99).  Predictor’s results on balanced dataset: MLP: Sensitivity(%)=80.56(3.15);Specificity(%)=88.6(2.13);Accuracy(%)=83.57(1.08). MLP stacked generalization: Sensitivity(%)=81.8(1.7);Specificity(%)=87.24(2.82);Accuracy(%)=83.86(0.55). Mixture of MLP-experts: Sensitivity(%)=82.06(0.87);Specificity(%)=87.76(0.96);Accuracy(%)=84.32(0.61). |
| Tang,2020 | 5-fold cross validation,  10-fold cross validation | No | Accuracy; Sensitivity; Specificity;  AUC;  F-score | No | Yes | MLP: Accuracy=73.14 ± 2.79;AUC=0.5376 ± 0.089;Sensitivity (%)= 99.71 ± 0.73;Specificity (%)=0.77 ± 2.32;F-score=0.844 ± 0.019;K=0.007 ± 0.025. MLPA: Accuracy=73.38 ± 2.94;AUC=0.6453 ± 0.055;Sensitivity (%)= 92.97 ± 6.27;Specificity (%)=19.51 ± 13.50;F-score=0.837 ± 0.023;K=0.146 ± 0.090. DT: Accuracy=67.41 ± 3.76;AUC=0.5683 ± 0.039;Sensitivity (%)= 91.10 ± 4.10;Specificity (%)=25.00 ± 9.83;F-score=0.833 ± 0.022;K=0.188 ± 0.089. SVM1: Accuracy=73.90 ± 2.14;AUC=0.5025 ± 0.009;Sensitivity (%)= 99.54 ± 1.18;Specificity (%)=0.95 ± 2.69;F-score=0.845 ± 0.014;K=0.007 ± 0.024. SVM2: Accuracy=73.46 ± 2.41;AUC=0.5034 ± 0.007;Sensitivity (%)= 99.34 ± 0.95;Specificity (%)=1.34 ± 2.09;F-score=0.846 ± 0.016;K=0.010 ± 0.020. SVM3: Accuracy=73.16 ± 2.16;AUC=0.5105 ± 0.015;Sensitivity (%)= 98.15 ± 2.41;Specificity (%)=3.95 ± 5.11;F-score=0.843 ± 0.014;K=0.028 ± 0.041. DNM: Accuracy=73.33 ± 2.48;AUC=0.6799 ± 0.042;Sensitivity (%)= 98.09 ± 2.52;Specificity (%)=4.82 ± 7.20;F-score=0.844 ± 0.016;K=0.037 ± 0.062. EDNM: Accuracy=75.14 ± 2.04;AUC=0.6818 ± 0.031;Sensitivity (%)= 93.81 ± 2.86;Specificity (%)=21.83 ± 9.11;F-score=0.848 ± 0.013;K=0.190 ± 0.074. |
| Hussain,2020 | 8-fold cross validation | No | Accuracy; Sensitivity; Specificity; | No | No | Step.Reg TRAI:FN =5867;TN =16131:FP =3224:TP =4945:Accuracy =0.69864;Sensitivity =0.45736;Specificity=0.83343. Step.Reg VALI:FN =4368;TN =12174;FP =2470;TP =3583;Accuracy =0.69737;Sensitivity =0.45064;Specificity=0.83133. Back.Reg TRAI:FN =6624;TN =16490;FP =2865;TP =4188;Accuracy =0.68545;Sensitivity =0.38735;Specificity=0.85198. Back.Reg VALI:FN =4815;TN =12564;FP =2080;TP =3136;Accuracy =0.69484;Sensitivity =0.39442;Specificity=0.85796. Forw.Reg TRAI:FN =6624;TN =16490;FP =2865;TP =4188;Accuracy =0.68545;Sensitivity =0.38735;Specificity=0.85198. Forw.Reg VALI:FN =4815;TN =12564;FP =2080;TP =3136;Accuracy =0.69484;Sensitivity =0.39442;Specificity=0.85796. Neural TR:FN =6124;TN =16409;FP =2946;TP =4688;Accuracy =0.69934;Sensitivity =0.43359;Specificity=0.84779. Neural VA:FN =4375;TN =12430;FP =2214;TP =3576;Accuracy =0.70839;Sensitivity =0.44975;Specificity=0.84881. Tree TRAI:FN =7469;TN =20477;FP =3270;TP =4907;Accuracy =0.70271;Sensitivity =0.39649;Specificity=0.8623. Tree VALI:FN =5527;TN =15491;FP =2485;TP =3589;Accuracy =0.70427;Sensitivity =0.3937;Specificity=0.86176. |

Abbreviation: SD=standard deviation; AUC=area calculated under the receiver operating characteristic (ROC) curve; TP=true positive; TN=true negative; FP=false positive; FN=false negative; PPV=negative predict value; ANN=artificial neural network; DT=decision trees; LR=logistic regression; NB=Naive Bayes; CI=confidence interval; FDTs=fuzzy decision trees; RCV=rate of change of some variables; RV=raw values; Mcc=Matthew’s correlation coefficient; NPV=Negative Predictive Value; FPR=False Positive Rate; FDR=False Discovery Rate; FNR=False Negative Rate; TPE=Tree of Parzen Estimators; MLP=multi-layer perceptron; BPNN=the back-propagated neural network; BN=Bayesian network; KNN=K-nearest neighbors; SVM=support vector machines; RBFNetwork=radial basis function network; SSL=semi-supervised learning; CHAID=CHi-squared Automatic Interaction Detection; CART=classification and regression tree; RF=random forest; DNN=deep neural network; MKL=multiple kernel learning; GB=Gradient Boosting; GAMOGB=genetic algorithm-based online gradient; OSELM=Online Sequential Extreme Learning Machine; OLRAB=Online Adaptive Boosting with the Adaptive Linear Regressor; OLRGB=On- line Gradient Boosting with the Adaptive Linear Regressor; OLR=online linear regressor; C-SVCF=C-Support Vector Classification Filter; SMOTE=Synthetic minority over-sampling technique; CSC=cost-sensitive classifier technique; PSO: particle swarm optimization; M imp=mean/mode imputation; EM imp=Expectation-Maximization imputation; KNN imp=KNN imputation; MI=multiple imputation; EFS=Ensemble Feature Selection; SFS=Stratified Feature Selection; GA=Genetic algorithm; LASSO=The Least Absolute Shrinkage and Selectionator operator; RUS=random under-sampling; GABC= gbest-guided artificial bee colony; EDNM=evolutionary dendritic neuron model; MLPA=the MLP with adaptive learning rate and momentum coefficient; SVM1=SVM trained by the radial basis function kernel; SVM2=SVM trained by the linear kernel; SVM3=SVM trained by the polynomial kernel; DNM=dendritic neuron model; PCRM=Parametric censored regression models; RSF= Random survival forests; BoostCI=Boosting concordance index; superPC=Supervised principal components regression; CNA=copy number alteration; mRMR=Max-Relevance and Min-Redundancy
