# Supplementary material for: Predicting breast cancer 5-year survival using machine learning: A systematic review
Source: PLoS One. 2021 Apr 16;16(4):e0250370. doi: 10.1371/journal.pone.0250370 (PMC8051758; doi:10.1371/journal.pone.0250370)
Supplement: S6 Table — (DOCX) [file pone.0250370.s006.docx]

**S6 Table. Candidate predictors used in survival prediction model and the important factors identified information of the 31 studies.**

| **First author, year** | **Number of Candidate predictors** | **Candidate predictors used in survival prediction model** | **Ranking candidate predictors** | **Rank of** **candidate predictors** |
| --- | --- | --- | --- | --- |
| Delen,2005 | 16 | (1)Race; (2)Marital status; (3)Primary site code; (4)Histology; (5)Behavior; (6)Grade; (7)Extension of disease; (8)Lymph node involvement; (9)Radiation; (10)Stage of cancer; (11)Site specific surgery code; (12)Age; (13)Tumor size; (14)Number of positive nodes; (15)Number of nodes; (16)Number of primaries | Yes | Sensitivity analysis on ANN output: Grade > Number of primaries > Stage of cancer > Radiation > Number of lymph nodes > Tumor size > Lymph node involvement > surgery > Number of positive lymph nodes > Behavior > Marital status > Age > Race > Histology > Extension of disease. |
| Bellaachia,2006 | 16 | (1)Race; (2)Marital status; (3)Primary site code; (4)Histologic type; (5)Behavior code; (6)Grade; (7)Extension of tumor; (8)Lymph node involvement; (9)Radiation; (10)Stage of cancer; (11)Site-specific surgery code; (12)Age; (13)Tumor size; (14)Number of positive nodes; (15)Number of Nodes; (16)Number of primaries | Yes | C4.5 DT information gain measure to rank the factors: Extension of tumor > Stage of cancer > Lymph node involvement > Site Specific Surgery > No of pos nodes > Tumor size > Histologic type > Age > Behavior code > Number of nodes > Grade > Marital status > Primary site > Radiation > Race > Number of primaries. |
| Endo,2008 | 10 | (1)Race; (2)Marital status; (3)Grade; (4)Reason of no surgery; (5)Radiation; (6)Radiation sequence with surgery; (7)Behavior Code ICD-O-3; (8)Age Record under 1 year olds; (9)SEER modified AJCC stage 3rded; (10)Number of primaries | No |  |
| Khan,2008 | 16 | (1)Race; (2)Marital Status; (3)Primary Site; (4)Histologic Type ICD; (5)Behavior Code; (6)Grade; (7)Extension of Tumor; (8)Node Involvement; (9)Site Specific Surgery; (10)Radiation; (11)Stage; (12)Age at Diagnosis; (13)Number of Positive Nodes; (14)Tumor Size; (15)Number of Nodes; (16)Number of Primaries | Yes | C4.5 decision tree information gain measure to rank the factors: Stage > Extension of Tumor > Node Involvement > Size Of Tumor > Site Specific Surgery > Number Of Positive Nodes > Age > Histologic Type > Marital Status > Behavior Code > Number Of Nodes > Grade > Primary Site > Radiation > Race > Number Of Primaries. |
| Thongkam,2008 | 11 | (1)Age; (2)Marital Status; (3) Occupation; (4)Basis of diagnosis; (5)Topography; (6)Morphology; (7)Extent; (8)Stage; (9)Received Surgery; (10)Received Radiation; (11)Received Chemotherapy | No |  |
| Choi,2009 | 14 | (1)Age at diagnosis; (2)AJCC stage 3rd edition; (3)Clinical extension of tumor; (4)Histologic type ICD 10; (5)Number of primary; (6)Site-specific surgery; (7)Scope of regional lymph node surgery; (8)Tis; (9)T4; (10)N0; (11)N1; (12)N2; (13)N3; (14)M3 | Yes | Sensitivity analysis on ANN model formulation: type of regional lymph node surgery > type of surgery > clinical extension of tumor > AJCC stage > age at diagnosis > histologic type ICD 10 > N3 > N0 > Tis > N1 > T4 > N2 > number of primaries >M1. |
| Liu,2009 | 16 | (1)Marital status; (2)Race; (3)Primary site code; (4)Grade; (5)Extension of disease; (6)Lymph node involve; (7)Radiation; (8)Stage of cancer; (9)First malignant; (10)Histology; (11)Site specific surgery code; (12)Age; (13)Tumor size; (14)Number of primaries; (15)Number of positive nodes; (16)Number of nodes | No |  |
| Wang,2013 | 9 | (1)Race; (2)Grade; (3)Extension of disease; (4) Site-specific surgery code; (5)Lymph node involvement; (6)Stage of cancer; (7)SEER modified AJCC stage 3rd; (8)Tumor size; (9)Number of positive nodes | No |  |
| Kim,2013 | 16 | (1)Tumor size; (2)Number of nodes; (3)Number of primaries; (4)Age at diagnosis; (5)Number of positive nodes; (6)Marital status; (7)Race; (8)Behavior code; (9)Grade; (10)Extension of tumor; (11)Node involvement; (12)Histological type according to the international classification of diseases (ICD); (13)Primary site; (14)Site-specific surgery; (15)Radiation; (16)Stage | No |  |
| Park,2013 | 16 | (1)Tumor size; (2)Number of nodes; (3)Number of primaries; (4)Age at diagnosis; (5)Number of positive nodes; (6)Marital status; (7)Race; (8)Behavior code; (9)Grade; (10)Extension of tumor; (11)Node involvement; (12)Histological type according to ICD; (13)Primary site; (14)Site-specific surgery; (15)Radiation; (16)Stage | No |  |
| Shin,2014 | 16 | (1)Tumor size; (2)Number of nodes; (3)Number of primaries; (4)Age at diagnosis; (5)Number of positive nodes; (6)Marital status; (7)Race; (8)Behavior code; (9)Grade; (10)Extension of tumor; (11)Node involvement; (12)Histological type according to ICD; (13)Primary site; (14)Site-specific surgery; (15)Radiation; (16)Stage | Yes | Lymph node involvement > Stage > Site-specific surgery > Number of positive nodes examined > Tumor size > Age at diagnosis > Extension of tumor > Number of nodes examined > Histological type ICD > Primary site > Grade, whereas ‘marital status’ and ‘race’ do not provide significant information. |
| Wang,2015 | 5 | (1)Age(A); (2)Tumor size(T); (3)Condition of tumor metastasizing to lymph nodes(N); (4)Whether the tumors had metastasized to other organs(M); (5)Surgery | Yes | According to the correlation coefficients: Tumor size (T) > Condition of tumor metastasizing to lymph nodes (N)> Whether the tumors had metastasized to other organs (M) > Surgery > Age (A). |
| Wang,2014 | 20 | (1)Race; (2)Marital status; (3)Primary site code; (4)Behavior Code ICD-O-3; (5)Grade; (6)Extension of disease; (7)Lymph node involvement; (8)Reason of no surgery; (9)Radiation; (10)Radiation sequence with surgery; (11)Histology; (12)Site-specific surgery code; (13)Stage of cancer; (14)SEER modified AJCC stage 3rd; (15)First malignant; (16)Age; (17)Tumor size; (18)Number of positive nodes; (19)Number of nodes; (20)Number of primaries | No |  |
| Chao,2014 | 7 | (1)Age; (2)Tumor size; (3)The number of examined lymph nodes; (4)The number of attacked lymph nodes; (5)Pathological staging; (6)Chemotherapy; (7)Radiotherapy | No |  |
| García-Laencina,2015 | 16 | (1)Age; (2)Tumor site; (3)Topography; (4)Contralateral; (5)Histological type; (6)Degree of differentiation; (7)T; (8)N; (9)M; (10)Stage; (11)Hormonal receptors; (12)HER2; (13)Surgery type; (14)Type of treatment; (15)Chemotherapy regime; (16)Hormonotherapy type | No |  |
| Lotfnezhad Afshar,2015 | 18 | (1)Race; (2)Marital status; (3)Primary site code; (4)Histology; (5)Behavior; (6)Grade; (7)Extension of tumor; (8)Lymph node involvement; (9)Radiation; (10)Stage; (11)Site specific surgery code; (12)ER Status; (13)PR Status; (14)Age; (15)Tumor size; (16)Number of positive nodes; (17)Number of nodes; (18)Number of primaries | Yes | In SVM model: Behavior > Lymph node involvement > Extension of tumor > Grade > Number of positive nodes > Age > Site specific surgery code > PR Status > Radiation > Stage;  BN: Grade > Histology > PR Status > Lymph node involvement > Site specific surgery code > ER Status > Race > Marital status > Number of nodes > Stage;  The CHi-squared Automatic Interaction Detection model: Extension of tumor > Number of positive nodes > Number of nodes > Tumor size > Behavior > ER Status > PR Status > Marital status > Age > Grade. |
| Khalkhali,2016 | 15 | (1)Primary site of tumor; (2)Metastases of tumor; (3)Behavior of tumor; (4)Grade of tumor; (5)Histology of tumor; (6)Stage of tumor; (7)Surgery of tumor; (8)Radiation of tumor; (9) HER2; (10)ER; (11)PR; (12)Age; (13)Tumor size; (14)Number of positive regional nodes; (15)Number of removed regional nodes | Yes | According to the sensitivity scores in rules: top candidate predictors of the rank: Stage, Number of positive regional nodes, Tumor size, HER2. |
| Shawky,2017 | 14 | (1)Marital status; (2) Race; (3) Birth Place; (4)Primary Site; (5)Laterality; (6)Histology; (7)Behavior; (8) Historic Stage; (9)Grade; (10)Age at diagnosis; (11)Year of birth; (12)#of primaries; (13)Regional Nodes Positive; (14)Tumor Size | No |  |
| Sun,2018 | 3 types | 25 Clinical features + 400 Genes from gene expression profile data + 200 Genes from CNA profile data | No |  |
| Sun,2018 | 5 types | 50 Gene expression + 10 CNA + 40 Methylation + 120 Protein + 130 Pathological image | No |  |
| Zhao,2018 | 27 | (1)Age at diagnosis; (2)NPI; (3)ER status; (4)PR status; (5)HER2 status; (6)Menopausal status; (7)Three-gene classifier subtype; (8)Degree of abnormality of cancer cells; (9)Primary tumor laterality; (10)Cellularity of tumor content; (11)Tumor size; (12)Tumor grade; (13)Tumor stage; (14)Breast surgery status; (15)Chemotherapy status; (16)Hormone therapy status; (17)Radiotherapy status; (18)Oncotree code , and1 genomic feature | Yes | On the whole, top candidate predictors of the rank: NPI, Age, Tumor stage, Size, ER status, PR status, HER2 status, Breast surgery status. |
| Fu,2018 | 23 | (1)Education; (2)Type of medical insurance; (3)Claim ratio of medical insurance; (4)Ki67; (5)CK5/6; (6)Molecular typing; (7)Neoadjuvant chemotherapy; (8)Postoperative chemotherapy; (9)Postoperative radiotherapy; (10)Type of endocrinotherapy; (11)AHT; (12)BMI; (13)Menopause age; (14)Number of births; (15)Age at first childbirth; (16)Duration between menarche and childbirth; (17)Duration between menarche and menopause; (18)T stage; (19)N stage; (20)Anatomical stage; (21)Prognosis stage; (22)Age at diagnosis; (23)Number of Ⅰ-Ⅱ lymph node metastases | Yes | Top candidate predictors of the rank: T stage, N stage and molecular typing, Type of endocrinotherapy, claim ratio of medical insurance, age at diagnosis. |
| Lu,2019 | 14 | (1)Race; (2)Marital status; (3)Primary site code; (4)Histology; (5)Behavior; (6)Grade; (7)Extension of disease; (8)Lymph node involvement; (9)Stage of cancer; (10)Site specific surgery code; (11)Age; (12)Tumor size; (13)Number of positive nodes; (14)Number of nodes | No |  |
| Abdikenov,2019 | 19 | (1)Age; (2)Race; (3)Year of birth; (4)Marital status; (5)State; (6)Year of diagnosis; (7)Behavior code; (8)Primary site; (9)Histologic type; (10)Grade; (11)Laterality; (12)Diagnostic confirmation; (13)Reason no surgery; (14)Tumor size; (15)Extension; (16)Lymph nodes; (17)Metastasis; (18)Cause of death; (19)Survival mouth | No |  |
| Kalafi,2019 | 23 | (1)Marital status; (2)Menopausal status; (3)Presence of family history; (4)Race; (5)Method of diagnosis; (6)Classification of breast cancer; (7)Laterality; (8)Cancer stage classification; (9)Grade of differentiation in tumor; (10)ER status; (11)PR status; (12)c-er-b2 status; (13)Primary treatment type; (14)Surgery status; (15)Type of surgery; (16)Method of axillary lymph node dissection; (17)Radiotherapy; (18)Chemotherapy; (19)Hormonal therapy; (20)Age; (21)Axillary lymph node; (22)Positive lymph nodes; (23)Tumor size | Yes | The five most important candidate factors: tumor size, stage, age, total axillary lymph node removed, and number of positive lymph nodes, such as breast cancer class (invasive or in-situ), family history, hormonal therapy, chemotherapy, radiotherapy, ER, PR, marital status, and method of axillary lymph node dissection do not affect the survivability prediction significantly. |
| Shouket,2019 | 10 | (1)Age; (2)Cancer tumor grade; (3)Specimen site distribution; (4)Specimen nature distribution; (5)Family history; (6)Tumor size; (7)Stages of disease; (8)Diagnosis type; (9)Homone receptor status; (10)HER2-neu status | No |  |
| Ganggayah,2019 | 23 | (1)Marital status; (2)Menopausal status; (3)Presence of family history; (4)Race; (5)Method of diagnosis; (6)Classification of breast cancer; (7)Laterality; (8)Cancer stage classification; (9)Grade of differentiation in tumor; (10)ER status; (11)PR status; (12)c-er-b2 status; (13)Primary treatment type; (14)Surgery status; (15)Type of surgery; (16)Method of axillary lymph node dissection; (17)Radiotherapy; (18)Chemotherapy; (19) Hormonal therapy; (20)Age; (21)Axillary lymph node; (22)Positive lymph nodes; (23)Tumor size | Yes | The six most important candidate factors: cancer stage classification, tumor size, total axillary lymph nodes removed, positive lymph nodes, primary treatment type, and method of diagnosis. |
| Simsek,2020 | 17 | (1)Cancer stage; (2)Nodes positive; (3)Grade; (4)Age; (5)Extension; (6)Primary site; (7)PR status; (8)ER status; (9)Tumor marker; (10)Surgery; (11)Radiation; (12)Race; (13)Tumor size; (14)Histology; (15)Marital status; (16)Behavior; (17)Lymph node  (In the final stage, based on GA and LASSO, 8, 10, and 11 variables were selected for 1-year, 5-year, and 10-year time points respectively) | Yes | 1-year: Age > Cancer stage > Tumor marker > Grade > Nodes positive > Extension > Lymph node;  5-year: Cancer stage > Tumor marker > Grade > PR status > Age > ER status > Nodes positive > Extension > Surgery > Lymph node;  10-year: Grade > Cancer stage > Nodes positive >Age > Tumor marker > PR status > Lymph node > ER status > Extension > Surgery. |
| Salehi,2020 | 35 | (1)Marital status; (2)Race; (3)Age; (4)Sequence number; (5)Primary site; (6)Laterality; (7)Histology ICD_O_3; (8)Behavioral ICD_O_3; (9)Grade; (10)Regional nodes positive; (11)Regional nodes examined; (12)CS tumor size; (13)CS extension; (14)CS lymph nodes; (15)CS mets; (16)RX surg prime site; (17)RX scope regional; (18)RX surg/reg; (19)Record ICD_9; (20)ER status; (21)PR status; (22)SEER stage; (23)First malignant prime; (24)SS 2000; (25)Prim by international rules; (26)SSF2; (27)SSF3; (28)SSF4; (29)SSF5 ; (30)SSF6 ; (31)AJCC stage group; (32)AJCC M; (33)AJCC T; (34)AJCC N; (35)Breast AJCC N | Yes | Age > AJCC T > AJCC M > AJCC Stage Group > ICD 9 primary > CS mets > Regional nodes positive > ER status > SEER degree > RX surg prime site > RX surg prime site > Regional nodes examined > First malignant prime > Behavior code > SS 2000 > SSF3 > IRAC > RX surg/reg > SSF2 > PR status > CS extension > Race > Breast AJCC N > Marital status > Histology type > AJCC N > Grade > Laterality > CS lymph nodes > Sequence number > SSF5,SSF4 > RX scope regional > CS tumor size > Primary site > SSF6 |
| Tang,2020 | 3 | (1)Age of patient at time of operation; (2)Patient's year of operation; (3)Number of positive axillary nodes detected | No |  |
| Hussain,2020 | 17 | (1)Race; (2)Marital status; (3)Primary site code; (4)Histology; (5)Behavior; (6)Sex; (7)grade; (8)extent of disease; (9)lymph node involvement; (10)radiation; (11)stage of cancer; (12)site-specific surgery code; (13)Age; (14)tumor size; (15)number of positive nodes; (16)number of nodes; (17)number of primaries | Yes | On the whole, the candidate predictors “SEER historic Stage A” is by far the most important predictor, followed by “size of tumor,” “Grade,” and “lymph node involvement new.” |

Abbreviation: ANN=artificial neural network; DT=decision tree; ER=Estrogen receptor; PR=Progesterone receptor; HER2=Human epidermal growth factor receptor 2; NPI=Nottingham Prognostic Index; AHT=Adjuvant hormonal therapy; ICD=the international classification of diseases; AJCC=American Joint Committee on Cancer; SSF=site-specific factor; CS=Collaborative Stage; BMI=Body Mass Index; SVM=support vector machines BN=Bayesian network; CNA=copy number alteration; GA=Genetic algorithm; LASSO=The Least Absolute Shrinkage and Selectionator operator;
